# Individual and Joint Effect of *Alpha*-Tocopherol and Hydroxytyrosol Acetate on the Oxidation of Sunflower Oil Submitted to Oxidative Conditions: A Study by Proton Nuclear Magnetic Resonance

**DOI:** 10.3390/antiox11061156

**Published:** 2022-06-13

**Authors:** Sofía del Caño-Ochoa, Ainhoa Ruiz-Aracama, María D. Guillén

**Affiliations:** Food Technology, Faculty of Pharmacy, Lascaray Research Center, University of the Basque Country (UPV-EHU), Paseo de la Universidad n 7, 01006 Vitoria-Gasteiz, Spain; sofia.delcano@ehu.eus (S.d.C.-O.); ainhoa.ruiz@ehu.eus (A.R.-A.)

**Keywords:** polyunsaturated edible oil, *alpha*-tocopherol, hydroxytyrosol acetate, binary mixtures and interactions, accelerated storage conditions, proton nuclear magnetic resonance, linoleic acyl group degradation rate, oxylipins formation, antioxidant–prooxidant effects

## Abstract

This study tackles the individual and joint effect of *alpha*-tocopherol and hydroxytyrosol acetate on the oxidation of sunflower oil submitted to accelerated storage conditions at intermediate temperature, in order to deepen the understanding of antioxidant–prooxidant behaviour. This was accomplished by ^1^H Nuclear Magnetic Resonance. For this purpose, the evolution of the degradation of both the main components of the oil and the aforementioned added compounds was monitored by this technique throughout the storage time. Furthermore, the formation of a very large number of oxylipins and the evolution of their concentration up to a very advanced stage of oil oxidation, as well as the occurrence of lipolysis, were also simultaneously studied. The results obtained show very clearly and thoroughly that in the oxidation process of the oil enriched in binary mixtures, interactions occur between *alpha*-tocopherol and hydroxytyrosol acetate that notably reduce the antioxidant effect of the latter compound with the corresponding negative consequences that this entails. The methodology used here has proved to be very efficient to evaluate the antioxidant power of mixtures of compounds.

## 1. Introduction

Antioxidant capacity is a very important attribute of certain food components. It prevents or delays the oxidation of food lipids which, as is well known, causes their degradation with the consequent loss of nutritional value and the generation of toxic compounds [1,2,3]. In addition, following food intake, compounds with antioxidant capacity can also act endogenously, preventing or delaying biological oxidation processes with important repercussions on human health [4].

For these reasons, much effort has been devoted to the search for sources of compounds capable of acting as antioxidants [5,6,7], and also to assessing the level of this capability. This ability has been matched to different capacities, such as that of scavenging free radicals, estimated by the DPPH (2,2-diphenyl-1-picrylhydrazyl) [8], and ABTS (2,2′-azino-*bis*(3-ethylbenzothiazoline-6-sulfonic acid) assays [9], or that of reducing ferric ions, FRAP [10], all of which are based on electron transfer (ET). Likewise, the antioxidant ability has also been matched with the capacity to absorb oxygen radicals (ORAC) [11,12] which is based on hydrogen atom transfer (HAT) [13]. However, the results provided by these methods are not always in agreement with each other, nor with the antioxidant activity demonstrated. This may be because these assays only cover a partial, albeit important, aspect of antioxidant capacity, and do not take into account other essential aspects that influence this ability.

It should be remembered that the antioxidant capacity of a compound depends not only on its nature and on the dose used, but also on other factors outside the compound that cannot be overlooked. Among these factors, the system in which the compound will prevent or delay the oxidation and the oxidative conditions to which it is subjected are essential, since it is known that, depending on them, a compound can present different levels of antioxidant power or even prooxidant capacity. Therefore, in order to attain reliable data on the antioxidant power of a compound in a given system when submitted to certain conditions, it is necessary to carry out a detailed and exhaustive monitoring of the evolution of the oxidation process that, under these conditions, undergoes the system not enriched and enriched in that compound at determined concentrations.

In agreement with the abovementioned, some studies aimed at estimating the antioxidant ability of different compounds have been made using, as systems able to be oxidized, pure compounds, such as methyl linoleate or triglycerides of different edible oils, submitted to certain oxidative conditions [14,15,16,17,18,19]. The results obtained with these lipid systems refer to them and cannot be extrapolated to other lipid systems. In these studies, the monitoring of the oxidation process was performed by estimating one or two parameters, considered to be oxidation markers. Among these are peroxide value, conjugated dienes [15,19,20,21,22], *p*-anisidine value [15,19], or the concentration of some oxidation compounds, measured at certain points of the process [14,16,17,23,24]. However, in some cases, these parameters have proven to be insufficient to adequately control the evolution of the oxidation process, leading, in some studies, to erroneous conclusions about the antioxidant capacity of the tested compound [22,25].

Although the aforementioned methods could provide some information on the antioxidant capacity of the compounds tested, this does not allow us to analyse the full effect of their presence on the oxidation process of the lipid system or to understand their behaviour. Recently, however, the development of a new methodology based on Proton Nuclear Magnetic Resonance, ^1^H NMR, allows, in a very simple way, the monitoring and study of the oxidation processes of oils, and of lipids in general, in an exhaustive and complete way. This makes it very suitable for the in-depth study of the antioxidant or prooxidant capacity [25,26,27,28,29,30,31]. The information provided by this methodology allows us to know the progression of the oxidation process in a global way from the point of view of the compounds degraded, and from the point of view of the oxylipins formed and their evolution. Thus, knowing to what extent and in what way the antioxidant or prooxidant compound influences the oxidation process and the formation of oxylipins can contribute not only to understanding its current behaviour, but also to anticipating its future behaviour.

It is not only interesting to know the effect of individual compounds with potential antioxidant capacity in different lipid systems, but also the effect of their mixtures. In fact, the behaviour of compounds in mixtures contributes to broadening the knowledge of the antioxidant or prooxidant capacity of each of the components. Moreover, in foods such as edible oils, vegetables and fruits, they are always forming mixtures, and it is to be expected that the antioxidant capacity of the mixture is conditioned and determined by the potential establishment of interactions between its components. In order to advance the knowledge of the capacity of binary mixtures to prevent or delay the oxidation of different lipid systems subjected to oxidative conditions, some studies have been carried out to investigate the possible interactions between their components and their effects on the overall antioxidant capacity of the mixture. In a general, summarized and simplistic way, it has been described that these interactions can lead to synergistic, additive or antagonistic effects [32,33]. However, most of the published studies on this topic were performed using electron transfer (ET) or hydrogen atom transfer (HAT) based assays [34], which as explained above have important limitations. Considering all of the above, and also the scarce correlations found between the presence of liposoluble compounds, supposedly with antioxidant power, and the actual antioxidant effect that they cause on edible oils [33,35,36], it seems evident that further studies are needed on the effect produced by mixtures of this kind of compound in lipid systems, in order to shed light on this complex issue.

In this context, the aim of this study is to deepen the understanding of the effect that the fortification of sunflower oil in *alpha*-tocopherol, αT, in hydroxytyrosol acetate, HTy-Ac, or in binary mixtures of them has on the evolution of all of them under accelerated storage conditions. The study was carried out using ^1^H NMR spectroscopy. *Alpha*-tocopherol has shown, in previous studies, to have antioxidant or prooxidant ability, depending on the lipid system and the experiment conditions [14,17,21,22,26,27,30,37,38,39]. Hydroxytyrosol acetate is a minor component of virgin olive oil with a well-recognized antioxidant ability [31,40,41,42,43,44]. The storage conditions used are oxidative and suitable for testing the antioxidant or prooxidant capacities of these compounds individually, and their binary mixtures, thus making it possible to analyse in-depth the potential interactions between these compounds and the effect of these interactions on the resulting antioxidant or prooxidant capacity. The study focuses on the evolution of the degradation of the main components of the oil, and of the added compounds throughout the storage process in oil samples enriched with these compounds individually and jointly. Simultaneously, in the same oil samples mentioned above, the formation of a large number of oxylipins, as well as the evolution of their concentration throughout the storage process is addressed. It is expected that the results obtained will provide valuable information that will contribute to a better understanding of the interactions between these compounds with potential antioxidant ability, as well as their effect, which may have an impact on food technology, nutrition and human health.

## 2. Materials and Methods

### 2.1. Samples of Study

The samples to be studied are sunflower oil, S, purchased from a local supermarket, and several samples of the same oil enriched with *alpha*-tocopherol, αT, with hydroxytyrosol acetate, HTy-Ac, or with binary mixtures of these compounds. The molar percentages of the different kinds of acyl groups of the sunflower oil, estimated from ^1^H NMR spectral data [45,46,47], are: 57.6 ± 0.3% of linoleic, 32.0 ± 0.7% of oleic and 10.4 ± 0.5% of saturated acyl groups. Although sunflower oil is one of the edible oils richest in *alpha*-tocopherol [48,49] the concentration of this tocopherol in this sunflower oil is not high enough to be detected by ^1^H NMR spectroscopy in the sample S.

The αT (98.2% purity) and HTy-Ac (99.54% purity) used to enrich the oil were purchased from Sigma-Aldrich (St. Louis, MO, USA) and Seprox Biotech (Madrid, Spain), respectively. The sunflower oil was enriched with αT at two different concentrations. The resulting samples were named S_5T_ and S_10T,_ in which the enrichment levels were 5 mmol and 10 mmol of αT, respectively, per mol of triglyceride (TG). The sunflower oil was also enriched with HTy-Ac at a concentration of 10 mmol HTy-Ac/mol TG and this sample was named S_10H_.

In addition, the sunflower oil was also enriched with two different binary mixtures of αT and HTy-Ac. These samples were named S_5T10H_ and S_10T10H_. In the former, the sunflower oil was enriched with (5 mmol αT + 10 mmol HTy-Ac)/mol TG and in the latter with (10 mmol αT + 10 mmol HTy-Ac)/mol TG.

### 2.2. Accelerated Storage Experiments and Study of the Samples Evolution by ^1^H NMR Spectroscopy

Aliquots of 10 g of the samples S, S_5T_, S_10T_, S_10H_, S_5T10H_ and S_10T10H_, were subjected to accelerated storage conditions at 70 °C, as in previous studies [50,51], until the samples were almost completely polymerized. The experiments were carried out in duplicate.

The evolution of each sample under the aforementioned conditions was monitored using ^1^H NMR spectroscopy. The equipment used was a Bruker Avance 400 spectrometer operating at 400 MHz. The operating conditions, the acquisition parameters, and the software employed were the same used in previous studies [50,51]. The spectrum of each sample was acquired in duplicate.

The identification of the compounds present in the different samples was carried out on the basis of the assignment of the ^1^H NMR signals to the hydrogen atoms of the different structures, like in previous studies [26,27,28,29,30,31,52], with the support of the standard compounds, indicated in Table S1 (Supplementary Material). These signals, their chemical shifts and their assignments to the various hydrogen atoms are given in Tables S2–S15 (Supplementary Material).

The estimation of the concentration of linoleic acyl group, of αT, and HTy-Ac, as well as of the several oxidation compounds present in the several samples over the storage time, in relation to that of triglycerides (TG) was carried out as in previous studies [29,30,31].

### 2.3. Statistical Analysis

The statistical analysis and graphical representations were performed using Microsoft Office Excel 2016, as in previous studies [30,31]. The statistical analysis was used for two purposes. Firstly, to calculate the standard deviations of the numerous determinations of the concentration of a very large number of compounds present in the oil samples throughout the accelerated storage. Secondly, to study the degradation pathways throughout the accelerated storage of the sunflower oil main component, of the *alpha*-tocopherol and of the hydroxytyrosol acetate added in the different samples.

## 3. Results and Discussion

The objective of this study, as mentioned above, is to elucidate the joint antioxidant or prooxidant effect of *alpha*-tocopherol, αT, and hydroxytyrosol acetate, HTy-Ac, in sunflower oil and, at the same time, to analyse whether this joint effect can be simply described as synergistic, additive or antagonistic [32,33,34] in relation to the effects caused by these compounds individually. Therefore, the results referring to the evolution of the oxidation process under accelerated storage conditions at 70 °C, of sunflower oil without enrichment in any of the mentioned compounds (S), or enriched in each of them (samples S_5T_, S_10T_, and S_10H_), or in any of their mixtures (samples S_5T10H_ and S_10T10H_), will be presented and discussed sequentially. These results will first concern the evolution of the degradation of both the main component of the oil, that is of the linoleic acyl group, and of the added compounds, that is of αT and HTy-Ac, during accelerated storage. They can provide important information on the overall evolution of the oxidation process in each sample and on the role played by the added compounds. After this, the formation of oxylipins in all samples will also be addressed, in order to analyse in-depth the joint effect of the added compounds both in the onset of their formation and in the evolution of their concentration throughout the storage time. Finally, the occurrence of lipolysis and 1,2-diglyceride formation during accelerated storage in all sunflower oil samples will also be addressed, in order to analyse the joint effect of binary mixtures on this issue.

### 3.1. Evolution of the Concentration of the Linoleic Acyl Group in the Different Sunflower Oil Samples over the Storage Time: Effect of Sunflower Oil Enrichment in αT, in HTy-Ac and in Binary Mixtures of αT and HTy-Ac

**Figure 1:**
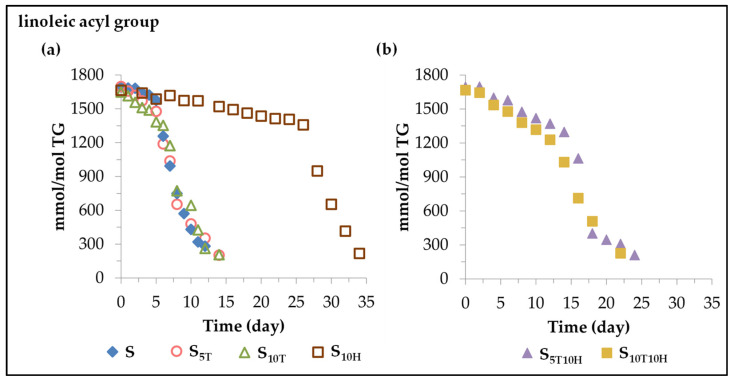
Evolution of the concentration, expressed as mmol of linoleic acyl group per mol of triglyceride (mmol/mol TG) over the storage time at 70 °C, up to a very advanced oxidation stage in: (**a**) samples S, S_5T_, S_10T_, and S_10H_; (**b**) samples S_5T10H_ and S_10T10H_.

The estimation of the concentration of the linoleic acyl group in the different oil samples during storage was performed as indicated in the experimental section from the ^1^H NMR spectral signal intensity, centred at 2.77 ppm, of the *bis*-allylic protons, shown in Table S2 (Supplementary Material), as described in previous studies [30,31,51]. The results obtained are depicted in Figure 1. The evolution of the concentration of the linoleic acyl group *versus* time fits quite well, in most cases, to two linear paths with different length and slope in each sample, shown in Table 1, and will be discussed below.

(*i*) *In sunflower oil sample S*. Sunflower oil subjected to accelerated storage conditions undergoes oxidation, resulting in the degradation of its components, the main one being the linoleic acyl group. As can be seen in Figure 1a and in the data of Table 1, the first stage of its degradation covers the first 5 days of storage, in which about 118 mmol of linoleic acyl group per mol of triglyceride (TG) are lost. After this stage, there is an acceleration in the degradation of this acyl group, so that in the second stage, which covers the following 7 days, there is an additional degradation of about 1300 mmol of linoleic acyl group per mol of TG, practically completing the entire oxidation process in a total of 12 days.

(*ii*) *In sunflower oil samples S_5T_ and S_10T_ enriched in αT*. As expected, the evolution of sunflower oil oxidation under accelerated storage conditions at 70 °C is affected by the enrichment of the oil in αT. As shown in Figure 1a and the data in Table 1, this causes an acceleration in the first stage of its degradation, compared to that which occurred in the non-enriched sample. Consequently, about 220 and 430 mmol of linoleic acyl group per mol of TG are lost in the S_5T_ and S_10T_ samples, respectively, in this first stage. Likewise, the second stage of the degradation of the acyl linoleic group is also affected by the enrichment of the oil in αT. This causes a slowing down of the degradation rate of this main component of sunflower oil (see Table 1), compared to its degradation rate in sample S, resulting in a lengthening of the whole oxidation process up to 14 days. These results are consistent with and reinforce previous results on the effect of the αT enrichment of other edible oils subjected to accelerated storage conditions [26,27,30].

(*iii*) *In sunflower oil in sample S_10H_ enriched in HTy-Ac*. According to the data represented in Figure 1a and given in Table 1, the enrichment of sunflower oil in HTy-Ac, in agreement with our previous results [32], causes a slowing down of the degradation rate of the linoleic acyl group in both stages of its degradation pathway, with respect to those of sample S. Due to this, the total oxidation process of sunflower oil, or in other words the total degradation of the linoleic acyl group in the S_10H_ sample, takes about 34 days, 26 of them corresponding to the first stage of degradation of the linoleic acyl group, in which only about 312 mmol are degraded, and the rest is degraded in the following 8 days, evidencing the delay caused by HTy-Ac in the oxidation of sunflower oil subjected to accelerated storage under the conditions of this study.

(*iv*) *In sunflower oil samples S_5T10H_ and S_10T10H_ enriched in binary mixtures of αT and HTy-Ac.* The evolution of the concentration of the linoleic acyl group in each of these sunflower oil samples also fits well to two linear paths whose data are represented in Figure 1b and shown in Table 1.

In both samples, the degradation rate in the first stage of the degradation of the linoleic acyl group is somewhat higher than that of the S sample. This shows that the presence of αT cancels the effect of HTy-Ac or vice versa, since each of these compounds has an opposite effect on the rate of degradation of the linoleic acyl group in this first stage of its degradation, as has been noted in points (ii) and (iii). Another variable also affected in this first stage of degradation is the duration of the stage, which is much longer in samples S_5T10H_ and S_10T10H_ than in samples S, S_5T_ or S_10T_, but shorter than in S_10H_. Consequently, in the first 12–14 days of accelerated storage, in which the first stage of linoleic degradation in samples S_5T10H_ and S_10T10H_ takes place, about 400–460 mmol of linoleic acyl group per mol of TG are degraded in these samples; however, in this same period of time in samples S, S_5T_ and S_10T_ the linoleic acyl group is almost completely degraded. It should be noted that although there is no great difference between the degradation rate and the duration of this first stage of degradation of the linoleic acyl group in samples S_5T10H_ and S_10T10H_, the effect of a higher enrichment in HTy-Ac than in αT in sample S_5T10H_ is slightly reflected in the variables mentioned above.

Furthermore, the effect of sunflower oil enrichment in binary mixtures of αT and HTy-Ac also affects the second stage of the linoleic acyl group degradation. The duration of this stage in samples S_5T10H_ and S_10T10H_ is longer than in the rest of the samples. This could be because the enrichment of sunflower oil in αT and HTy-Ac individually (samples S_5T_, S_10T_ and S_10H_) also causes, in both cases (see data in Table 1), a lower degradation rate of the linoleic acyl group in this second stage than that observed in sample S, and in this case the effects of both αT and HTy-Ac go in the same direction. For this reason, although in samples S_5T10H_ and S_10T10H_ most of the linoleic acyl group is degraded during this second stage, this occurs more slowly than in the rest of the samples.

As a summary, it can be said that, considering that the linoleic acyl group is not only the main component of sunflower oil, but also the most prone to be oxidized, the evolution of its degradation can be considered a very good representation of the evolution of the oxidation of sunflower oil. This study shows the complexity of the effects that the tested binary mixture provokes in the evolution of the oxidation process of sunflower oil submitted to accelerated storage. Taking as a reference the oxidation process in sample S, the αT enrichment of the oil accelerates the process in the first stage, while the HTy-Ac enrichment slows it down. However, in the second stage of the process, the enrichment in either of them slows it down. The enrichment of the oil in both binary mixtures (samples S_5T10H_ and S_10T10H_) causes, in the first stage of the oxidation process, an oxidation rate lower than that caused by the enrichment in αT (samples S_5T_ and S_10T_), but higher than that observed in sample S. This evidences the predominant effect of αT *versus* that of HTy-Ac, at both concentrations in the binary mixtures, in this first stage of the process. However, in the second stage of the oxidation process, as the individual enrichment of the oil in αT or in HTy-Ac slows down the process in relation to that of sample S, the joint effect of both compounds in the binary mixtures causes a greater slowing down of the process than that produced by these compounds individually. As a result, this second stage of the process is longer in samples S_5T10H_ and S_10T10H_ than in the rest of the samples.

The set of variables, oxidation rate at each stage, duration of each stage and amount of linoleic acyl group degraded at each stage, provides an overall picture of the oxidation progress of sunflower oil during accelerated storage. All these variables are governed by the presence of αT and HTy-Ac in the oil sample and the evolution of the concentration of the latter in the oxidation process can also shed complementary light to obtain a deeper understanding of the global evolution of the sunflower oil oxidation process under the conditions of this study.

### 3.2. Evolution of the Concentration of αT and HTy-Ac in the Different Sunflower Oil Samples Enriched in These Compounds over Accelerated Storage Time

**Figure 2:**
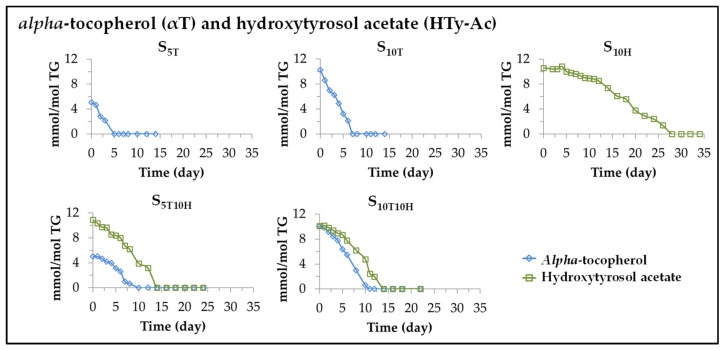
Evolution of the concentration, expressed as mmol/mol TG, of *alpha*-tocopherol, αT, and of hydroxytyrosol acetate, HTy-Ac, in sunflower oil enriched in these compounds individually (samples S_5T_, S_10T_ and S_10H_), or in binary mixtures of them (samples S_5T10H_ and S_10T10H_), throughout the accelerated storage at 70 °C up to a very advanced oxidation stage.

It is known that when edible oils are subjected to oxidative conditions, degradation of both its major and minor components occurs, whether the latter are natural or added, and regardless of whether they behave as antioxidants or prooxidants [25,26,27,30,31,49,53]. Therefore, under accelerated storage conditions, simultaneously with the degradation of the main components of the oil, the degradation of the added αT and HTy-Ac will also occur. The evolution of these degradations can be followed from the data provided by the ^1^H NMR spectra of the aforementioned samples, since these compounds have some protons giving specific spectral signals, shown in Table S3 (Supplementary Material) not overlapping with others. Therefore, from the intensity of these signals in the different spectra, the evolution of αT and HTy-Ac concentration in the different sunflower oil samples can be estimated over the storage time. The results obtained are depicted in Figure 2. It can be observed that the evolution of the concentration of these compounds quite adequately matches one or two linear stages whose slopes are shown in Table 2, together with the correlation coefficients of these linear stages given in brackets. The slopes of these lines coincide with the degradation rates of these compounds at each degradation stage of the different samples. The results obtained in each sample will be discussed below.

(*i*) *Evolution of the concentration of αT in sunflower oil samples S_5T_ and S_10T_.* As shown in Figure 2 and Table 2, the degradation of this compound starts from the beginning of storage, and the storage time at which it is totally depleted depends on its initial concentration in the oil, occurring later the higher the enrichment of the oil in this compound (day 5 at S_5T_ and day 7 at S_10T_). Moreover, the total depletion of αT coincides approximately with the change in the degradation rate of the linoleic acyl group (see Table 1), evidencing the decisive role of this compound in the degradation of the oil main component and therefore in the oxidation of sunflower oil.

Furthermore, the higher the initial concentration of αT in the oil sample, the higher its degradation rate (see Table 2). This compound accelerates the degradation of the linoleic acyl group in its first stage of degradation in relation to the non-enriched sample S, that is, it behaves as a prooxidant, as demonstrated in previous studies [26,27,30,54]. In addition, and although this compound is absent in the second degradation stage of the linoleic acyl group, the initial enrichment of the oil in it slows down the rate of degradation of the linoleic acyl group in this second stage, compared to that of the non-enriched sample S (see Table 1).

(*ii*) *Evolution of the concentration of HTy-Ac in sunflower oil sample S_10H_*. This compound behaves as an antioxidant in the S_10H_ sample subjected to accelerated storage conditions, as expected [31,40,41,43,44,55], and degrades during the process. Its degradation path fits well to two linear stages. As Table 2 shows, during the first stage, lasting about 12 days, the degradation rate of HTy-Ac is very small. In the second stage, lasting about 16 days, the degradation rate of this compound is higher than that of the first stage, but both are much lower than that of αT in the S_10T_ sample. The differences in the degradation rates of αT and HTy-Ac are very noticeable, as shown in Figure 2 and Table 2. The consequence of these differences in degradation rate is that HTy-Ac does not fully degrade in the S_10H_ sample until after 28 days under accelerated storage conditions, while αT disappears in the S_10T_ sample after 7 days under the same conditions. It is observed that as in the samples enriched in αT, there is a coincidence between the time at which HTy-Ac is totally depleted and the time at which the degradation rate of the linoleic acyl group undergoes an important change (see Figure 1 and Figure 2 and Table 1 and Table 2), evidencing the importance of this compound in the evolution of the degradation of the linoleic acyl group, and in turn in the evolution of the oxidation process of the sunflower oil. It is also worth noting that, even once HTy-Ac is fully degraded, in the second stage of the oxidation process of the S_10H_ sample, its influence is evidenced by slowing down the degradation rate of the linoleic acyl group, compared to that of the second stage of degradation of this same group in the S sample.

(*iii*) *Evolution of the concentration of αT and of HTy-Ac in sunflower oil samples S_5T10H_ and S_10T10H_*. When these samples are subjected to accelerated storage, as shown in Figure 2 and Table 2, the degradation rate of αT is lower in both samples S_5T10H_ and S_10T10H_ than that observed in samples S_5T_ and S_10T_, and as a consequence, αT is present in the former samples for up to about 8–10 days under accelerated storage conditions, *versus* 5 and 7 days in samples S_5T_ and S_10T_, respectively. However, the degradation rate of HTy-Ac in the two stages of its degradation process in samples S_5T10H_ and S_10T10H_ is considerably higher than that observed in sample S_10H_, and as a consequence HTy-Ac is present in the former samples for approximately half as long as in the last sample. In addition, the time at which HTy-Ac is completely depleted coincides with the change in the rate of the degradation of the linoleic acyl group (see Figure 1 and Figure 2 and Table 1 and Table 2).

In short, in the samples enriched exclusively in αT, this degrades very rapidly at both concentrations, while the degradation rate of HTy-Ac in the samples enriched exclusively in this compound is almost four times lower than that of αT. In the sunflower oil samples enriched in binary mixtures, the results obtained demonstrate the existence of interactions between HTy-Ac and αT, since the degradation rate of αT decreases somewhat, while that of HTy-Ac increases considerably, compared to those observed in the samples enriched in each of these compounds individually. This suggests that the antioxidant capacity of HTy-Ac inhibits, up to a certain level, the degradation of αT by delaying it, and as a consequence the degradation of HTy-Ac is accelerated so that the antioxidant capacity exhibited by this compound in the oxidation of sunflower oil is reduced in relation to that shown by this compound when the oil is enriched exclusively in it. In other words, it could be said that HTy-Ac, the strong antioxidant, regenerates to some extent αT, the prooxidant, and as a consequence the antioxidant capacity of the former is reduced due to its oxidation. Moreover, it has been observed in all samples that the time at which the degradation rate of the linoleic acyl group changes coincides with the total disappearance of αT and HTy-Ac in the samples enriched individually in these compounds, and with the disappearance of HTy-Ac in the samples enriched in the binary mixtures of αT and HTy-Ac, evidencing the important role of these compounds in the evolution of oil oxidation.

In any oxidation process, the formation of new compounds occurs simultaneously with the degradation of other ones. In order to have a complete view of the effect that the enrichment of sunflower oil in the aforementioned compounds and their mixtures has on its oxidation process, when it is subjected to accelerated storage conditions, it is also necessary to study the formation of oxylipins and their evolution in this process.

### 3.3. Evolution of the Concentration of the Different Oxylipins Formed throughout the Accelerated Storage in the Different Sunflower Oil Samples: Effect of the Enrichment of Sunflower Oil in αT and HTy-Ac and in Binary Mixtures of These Compounds

As is well known, the degradation of the main components of edible oil leads to the formation of oxidation compounds, some of which are well known, such as hydroperoxides, also called primary oxidation compounds, and secondary or further oxidation compounds, such as hydroxy-, keto- and epoxy-derivatives, as well as different types of aldehydes and other derived compounds. The formation of many of these compounds and the evolution of their concentration throughout the accelerated storage was monitored by ^1^H NMR spectroscopy simultaneously with the evolution of linoleic acyl group and that of the added compounds αT and HTy-Ac discussed above. Using the aforementioned analytical tool and the methodology indicated in the experimental section, a large number of oxylipins were identified and quantified in the different sunflower oil samples. Their identification, as in previous studies [26,27,28,29,30,31,52] was possible by the appearance in the spectra of specific signals of some of their protons, indicated in Tables S4–S15 (Supplementary Material). Using the intensity of these signals, the concentration of the oxylipins detected in the different sunflower oil samples over the accelerated storage time was determined and represented in the figures that will be shown later on.

The oxylipins found have been grouped into three groups. And the effect of sunflower oil enrichment in αT, HTy-Ac and binary mixtures of αT and HTy-Ac on the onset of their formation and on the evolution of their concentration will be analysed in each group. In addition, it should be remembered that harmful effects on human health have been attributed to some of these compounds [56,57,58,59,60,61,62,63,64,65,66,67,68,69,70,71,72,73,74,75,76,77,78,79,80,81], so the influence that the enrichment of the oil in αT, HTy-Ac and binary mixtures of them has on the formation of these compounds is a subject of great interest.

#### 3.3.1. Long Chain Oxylipins with Origin in the Peroxidation of the Linoleic Acyl Group

**Figure 3:**
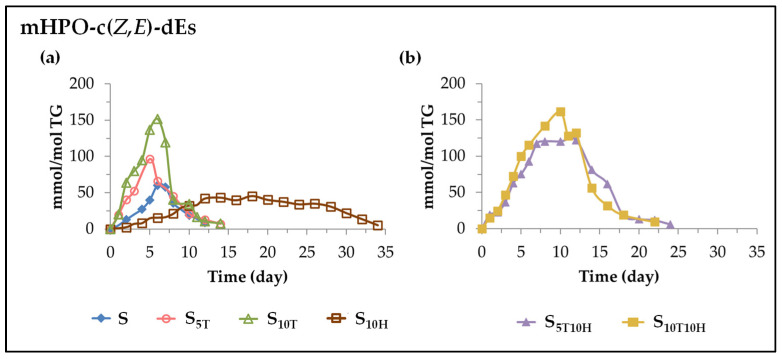
Evolution of the concentration, expressed in mmol/mol TG, over the accelerated storage time at 70 °C, up to a very advanced oxidation stage, of mHPO-c(*Z*,*E*)-dEs in: (**a**) S, S_5T_, S_10T_, and S_10H_ samples; (**b**) S_5T10H_ and S_10T10H_ samples.

This group includes all those oxylipins bearing one or two oxygen atoms whose origin is in the peroxidation of the linoleic acyl group [82,83,84,85] and which maintain the same chain length as their precursor without introducing branches in it, and this will be addressed below. They could also be formed endogenously by the action of lipooxygenases on the linoleic group [86].

(A) *Evolution of the concentration of monohydroperoxy conjugated Z*,*E-dienes (mHPO-c(Z*,*E)-dEs)*. These oxylipins are the first to form when sunflower oil is subjected to accelerated storage. The onset of their formation and the evolution of their concentration in the different samples are shown in Figure 3. As the oil and the oxidative conditions are the same in all cases, the differences found between the samples on the formation of these oxylipins are due exclusively to the effect of their different enrichment in αT, in HTy-Ac or in binary mixtures of αT and HTy-Ac. These differences are very noticeable.

(*i*) *In sunflower oil. Sample S.* As can be observed in Figure 3a, the formation of mHPO-c(*Z*,*E*)-dEs in S sample starts very early and their concentration increases very rapidly, reaching its maximum value (about 60 mmol/mol TG) after 6 days under accelerated storage conditions, after which it decreases sharply to very low values around day 12. The evolution of the mHPO-c(*Z*,*E*)-dEs concentration in this sample can be summarized in the two stages mentioned above with a very short time spent at the maximum value reached (see Figure 3a).

(*ii*) *In sunflower oil enriched in αT. Samples S_5T_ and S_10T_.* The formation of mHPO-c(*Z*,*E*)-dEs in these samples is earlier and occurs at a higher rate than in S sample (see Figure 3a) with these differences being more noticeable the greater the αT enrichment. This evidences the behaviour of αT as a prooxidant, in agreement with what has been observed in previous studies in other edible oils [26,27,30,54]. These oxylipins reach their maximum concentration (about 100 mmol/mol TG in S_5T_ and about 160 mmol/mol TG in S_10T_) after 5–6 days under accelerated storage conditions and they degrade very rapidly. The evolution of their concentration in samples S_5T_ and S_10T_, as shown in Figure 3a, could also be described as formed by two stages, one of increasing concentration and the other of decreasing concentration. It is noticeable that the onset of the decrease in the concentration of mHPO-c(*Z*,*E*)-dEs coincides in time with the total disappearance of αT in these samples and with the change in the rate of degradation of the linoleic acyl group, demonstrating the important role also played by αT in the formation and degradation of mHPO-c(*Z*,*E*)-dEs.

(*iii*) *In sunflower oil enriched in HTy-Ac. Sample S_10H_*. The effect of the sunflower oil enrichment in HTy-Ac on the formation of mHPO-c(*Z*,*E*)-dEs under accelerated storage conditions is very different from that produced by the αT enrichment. In the sunflower sample S_10H_, as Figure 3a shows, the formation of mHPO-c(*Z*,*E*)-dEs occurs later and also at a slower rate than in the rest of the samples, revealing the behaviour of HTy-Ac as an antioxidant, in agreement with previous studies [31,41,42,43]. Therefore, mHPO-c(*Z*,*E*)-dEs not only reach a lower maximum concentration (less than 50 mmol/mol of TG) than in the other sunflower samples, but they also reach it later (after about 12 days under storage conditions). It is very remarkable that this period of 12 days coincides with the first stage of the HTy-Ac degradation path and it is also worth noting that, despite the important role developed by this compound in slowing down the degradation of the linoleic acyl group and the formation of mHPO-c(*Z*,*E*)-dEs over these 12 days, its degradation rate, as shown in Figure 2 and Table 2, is very low. In addition, it should also be noted that the concentration of mHPO-c(*Z*,*E*)-dEs remains stable for a very long period of time (from day 12 to about day 28 of accelerated storage), as Figure 3a shows, after which it starts to decrease. This period of nearly 16 days (between days 12 and 28) agrees with the second stage of the HTy-Ac degradation path. From the data in Table 2 and those depicted in Figure 3a, it seems evident that the maintenance of stabilization of mHPO-c(*Z*,*E*)-dEs concentration in the S_10H_ sample from day 12 to day 28 implies a higher rate of HTy-Ac degradation than that required for the slowing down of the rate of mHPO-c(*Z*,*E*)-dEs formation in the first 12 days of accelerated storage. In summary, the evolution of the concentration of these oxylipins in the S_10H_ sample could be described as consisting of three stages, a growth stage, a longer maintenance stage and a decreasing stage. Furthermore, it has also been observed that, as in samples S_5T_ and S_10T_, in sample S_10H_, the onset of the decrease of the concentration of mHPO-c(*Z*,*E*)-dEs, the change in the degradation rate of the linoleic acyl group, and the total HTy-Ac depletion occur at, approximately, the same storage time. To our knowledge, this is the first time that the aforementioned coincidence in time between these three important milestones in the oxidation process of an edible oil in the presence of antioxidants or added prooxidants has been demonstrated. The above results also prove the power of αT and HTy-Ac to influence both the degradation of major oil components and the formation of mHPO-c(*Z*,*E*)-dEs.

(*iv*) *In sunflower oil enriched in binary mixtures of αT and HTy-Ac. Samples S_5T10H_ and S_10T10H_*. The enrichment of sunflower oil in binary mixtures of αT and HTy-Ac also has an important influence on the formation of these oxylipins, as shown in Figure 3b. Their formation starts at the beginning of accelerated storage, as in samples S_5T_ and S_10T_, and the end of the first stage of their evolution lasts approximately until the complete depletion of αT, which is delayed with respect to that of the samples enriched exclusively in αT (see Figure 2 and Figure 3). The formation rate of mHPO-c(*Z*,*E*)-dEs in this first stage is lower than in these latter samples, but much higher, not only than in S_10H_, but also than in S. It could be said that in this first stage, as a consequence of the interactions between αT and HTy-Ac, the prooxidant effect predominates over the antioxidant effect, in agreement with what was previously observed regarding the degradation rate of the linoleic acyl group in the same samples and oxidation stage. The concentration reached by mHPO-c(*Z*,*E*)-dEs at the end of this first stage is even higher than that reached in the S_5T_ and S_10T_ samples. This could be due to the antioxidant and regenerative effect of HTy-Ac on αT, which would explain why the duration of this first stage increases in these samples with respect to that of the samples enriched only in αT, and decreases with respect to that of the samples enriched in HTy-Ac as shown in Figure 3. It could be said that, up to a certain level, HTy-Ac inhibits the degradation of αT by delaying it, and as a consequence, the degradation of HTy-Ac is accelerated, so the antioxidant effect on sunflower oil is reduced in relation to that observed in the sample enriched exclusively in HTy-Ac, S_10H_.

After the first stage, the evolution of the concentration of these oxylipins in sample S_5T10H_ is somewhat different from that of sample S_10T10H_. In sample S_5T10H_, as in sample S_10H_ (see Figure 3), there is a second stage in which stabilization of the concentration of these oxylipins occurs. The similarity between the evolution of the concentration of these oxylipins in these last two samples could be because in both samples, at the end of the first stage, there is still a considerable concentration of HTy-Ac (see Table 2). However, this is higher in sample S_10H_ than in sample S_5T10H_, and possibly because of this, this second stage is longer in the first than in the second sample, as shown in Figure 3. After this second stage, which lasts until about day 12 of storage, a third stage begins, coinciding with the onset of the decrease of the concentration of mHPO-c(*Z*,*E*)-dEs (see Figure 3b), with a very low concentration of HTy-Ac (see Figure 2 and Table 2), and with the change in the degradation rate of the linoleic acyl group (see Table 1 and Figure 1).

Nevertheless, in sample S_10T10H_, the first stage lasts until about day 10–11 of storage, at the end of which the mHPO-c(*Z*,*E*)-dEs reach the maximum concentration, and there is no stabilization stage of the concentration of these oxylipins as that observed in sample S_5T10H_. This could probably be due to the concentration of HTy-Ac at the end of the first stage in sample S_10T10H_ (see Table 2) not being sufficient to produce the abovementioned stabilization of the concentration. Therefore, in this sample, the decrease of the concentration of mHPO-c(*Z*,*E*)-dEs begins sooner than in sample S_5T10H_ (see Figure 3b).

**Figure 4:**
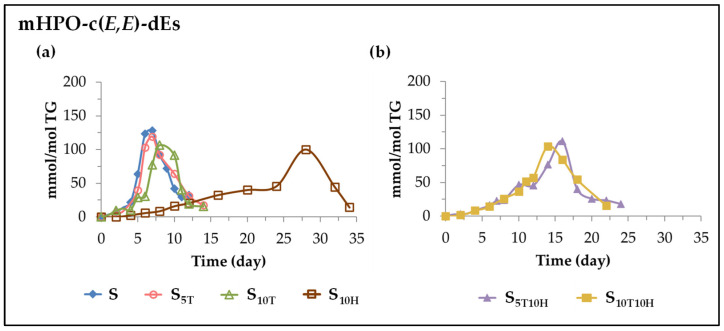
Evolution of the concentration, expressed in mmol/mol TG, over the storage time at 70 °C, up to a very advanced oxidation stage, of mHPO-c(*E*,*E*)-dEs in: (**a**) S, S_5T_, S_10T_, and S_10H_ samples; (**b**) S_5T10H_ and S_10T10H_ samples.

In summary, in the sunflower oil samples enriched in binary mixtures of αT and HTy-Ac, the formation of mHPO-c(*Z*,*E*)-dEs begins much earlier than in the sample enriched exclusively in HTy-Ac, showing the predominance in this first stage of the prooxidant effect *versus* the antioxidant one, in agreement with what was observed in the degradation rate of the linoleic acyl group in these samples. This is also evidenced by the fact that these oxylipins reach even higher maximum concentrations in the samples enriched in binary mixtures of αT and HTy-Ac than in the samples enriched in αT alone. The presence of HTy-Ac contributes to a certain slowing down of the rate of formation of these oxylipins, compared to that of the samples enriched only in αT, as well as to a stabilization of their concentration, which is greater the higher the ratio between the concentrations of HTy-Ac and αT in the sample. Consequently, in binary mixtures, the effect of HTy-Ac contributes to the extension of the period in which the mHPO-c(*Z*,*E*)-dEs remain undegraded, but it is much shorter than in the sample enriched exclusively in HTy-Ac. These results show the important negative effect produced by αT in the binary mixtures on the oxidative stability of sunflower oil under the conditions of this study and, as a consequence, on the formation of these oxylipins and others derived from them. The fact that the maximum concentration reached by mHPO-c(*Z*,*E*)-dEs in samples S_5T10H_ and S_10T10H_ is somewhat higher than that reached in samples S_5T_ and S_10T_ suggests that HTy-Ac regenerates αT to some extent.

(B) *Evolution of the concentration of monohydroperoxy conjugated E*,*E-dienes (mHPO-c(E*,*E)-dEs)*. These oxylipins are also included among the so-called primary oxidation compounds. They have been described as being formed by the isomerization of mHPO-c(*Z*,*E*)-dEs [87,88,89]. The formation and evolution of these oxylipins over storage time is also considerably affected by the enrichment of sunflower oil in αT and HTy-Ac or in their binary mixtures.

(*i*) *In sunflower oil. Sample S.* As can be observed in Figure 3a and Figure 4a in this sample, the onset of the formation of mHPO-c(*Z*,*E*)-dEs and mHPO-c(*E*,*E*)-dEs coincides, as does the storage time at which these oxilypins reach the maximum concentration. However, the maximum concentration reached by mHPO-c(*E*,*E*)-dEs (about 130 mmol/mol TG) is higher than that reached by mHPO-c(*Z*,*E*)-dEs (about 60 mmol/mol TG), in agreement with the results of previous studies on other edible oils [25,26,27,29,31]. This is because the isomerization of *Z*,*E* to *E*,*E* is clearly favoured by the storage temperature (70 °C), since at lower temperatures both types of oxylipins reach similar maximum concentrations [30].

(*ii*) *In sunflower oil enriched in αT. Samples S_5T_ and S_10T_.* The enrichment of sunflower oil in αT, as shown in Figure 4a, leads to a delay in the onset of formation and a slowing down of the rate of formation of mHPO-c(*E*,*E*)-dEs, relative to those occurring for the same oxylipins in the unenriched sample S. As a consequence, lower maximum mHPO-c(*E*,*E*)-dEs concentrations are reached in the enriched samples than in S, and this maximum is reached later. It is also observed in the comparison of Figure 3a and Figure 4a that the enrichment in αT causes a delay in the formation onset of these oxylipins and a slowing down of their rate of formation with respect to that of mHPO-c(*Z*,*E*)-dEs in the same samples, with both differences being greater the higher the enrichment in αT. As a consequence, in the sample most enriched in αT, S_10T_, the mHPO-c(*E*,*E*)-dEs reach lower maximum concentrations than in the S and S_5T_ samples and this maximum is reached later, in contrast to what happened in the evolution of the mHPO-c(*Z*,*E*)-dEs concentration. Despite this, the total concentration reached by mHPO-c-dEs is higher the higher the enrichment level in αT. These results are consistent with and reinforce previous results from studies on other edible oils [26,27,30]. In short, it seems evident that enrichment of the oil in αT produces opposite effects with respect to the formation of mHPO-c(*Z*,*E*)-dEs and mHPO-c(*E*,*E*)-dEs, accelerating the onset of formation and increasing the rate of formation of the former with respect to that of the unenriched sample S, as well as delaying the onset of formation and decreasing the rate of formation of the latter to a greater extent, the higher the level of enrichment in αT.

(*iii*) *In sunflower oil enriched in HTy-Ac. Sample S_10H_*. The enrichment of sunflower oil in HTy-Ac produces a much longer delay in the onset of the formation of mHPO-c(*E*,*E*)-dEs than that produced by αT, and a prolonged stage in which the rate of formation of these compounds increases very slowly. This stage lasts until the concentration of HTy-Ac in the sample is almost totally depleted, after which there is a sharp increase in their rate of formation that also coincides with the decrease in the concentration of mHPO-c(*Z*,*E*)-dEs from which they derive [89,90]. These oxylipins reach their maximum concentration when HTy-Ac is fully depleted and when the rate of degradation of the linoleic acyl group changes, as shown in Figure 1, Figure 2, Figure 3a and Figure 4a. The maximum concentration reached (about 100 mmol/mol TG) is somewhat lower than that reached in sample S_10T_.

(*iv*) *In sunflower oil enriched in binary mixtures of αT and HTy-Ac. Samples S_5T10H_ and S_10T10H_*. Considering all of the above, it is to be expected that the formation of mHPO-c(*E*,*E*)-dEs in sunflower oil enriched in binary mixtures of αT and HTy-Ac will also be considerably affected by the interactions between these compounds. The onset of their formation starts later than in the samples enriched in αT, but slightly earlier than in the sample enriched in HTy-Ac, as shown in Figure 4b. The evolution of their concentration, is, to some extent similar to what occurred in the S_10H_ sample. This increases very slowly until the HTy-Ac concentration becomes very small and the mHPO-c(*Z*,*E*)-dEs begin to degrade, after which the mHPO-c(*E*,*E*)-dEs concentration begins to increase very sharply, reaching the maximum value when the total depletion of HTy-Ac is produced (14 days of storage) and when the rate of degradation of the linoleic acyl group changes, as Figure 1, Figure 2, Figure 3b and Figure 4b show. The maximum concentration reached by these oxylipins is somewhat higher than that reached in S_10H_, and after about 25 days of storage these oxylipins are completely degraded, leading to the formation of others.

**Figure 5:**
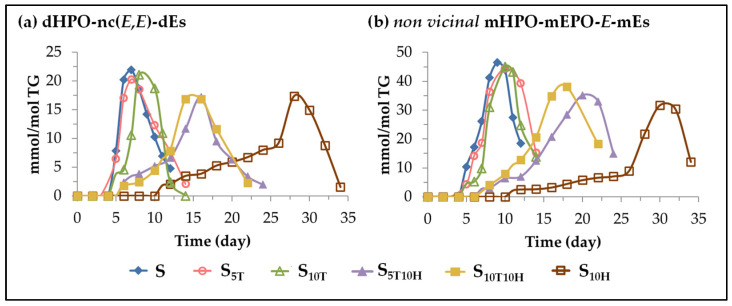
Evolution of the concentration, expressed in mmol per mol of triglyceride (mmol/mol TG), in samples S, S_5T_, S_10T_, S_10H_, S_5T10H_ and S_10T10H_, over the storage time at 70 °C, up to a very advanced oxidation stage, of: (**a**) dHPO-nc(*E*,*E*)-dEs; (**b**) *non-vicinal* mHPO-mEPO-*E*-mEs.

Considering that a significant number of oxylipins are derived from the aforementioned primary oxidation compounds [82,83,84,85], it would be expected that the effect of oil enrichment in αT, in HTy-Ac and in binary mixtures of αT and HTy-Ac on the formation and evolution of the concentration of mHPO-c-dEs would also be reflected in those of their derived oxylipins. Therefore, the possibility that the onset of formation of the latter oxylipins and the evolution of their concentration during accelerated storage can be predicted from those observed in mHPO-c-dEs will also be contemplated.

(C) *Evolution of the concentration of other oxylipins bearing hydroperoxy group*. This subgroup includes dihydroperoxy non-conjugated *E*,*E*-dienes (dHPO-nc(*E*,*E*)-dEs) and *non-vicinal* monohydroperoxy monoepoxy *E*-monoenes (*non-vicinal* mHPO-mEPO-*E*-mEs). Both types of compounds are considered to be derivatives of mHPO-c(*Z*,*E*)-dEs [71,85,91,92,93,94,95], and in turn have been considered as precursors of different further oxidation compounds [94,95,96,97,98]. The evolution of their concentration in sunflower oil not enriched or enriched in αT, in HTy-Ac and in binary mixtures of αT and HTy-Ac, throughout the accelerated storage, is shown in Figure 5.

**Figure 6:**
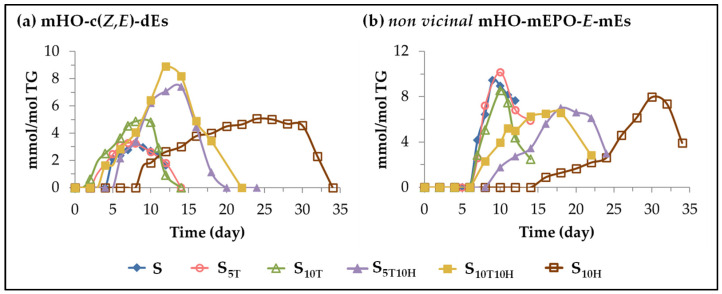
Evolution of the concentration, expressed in mmol per mol of triglyceride (mmol/mol TG), in samples S, S_5T_, S_10T_, S_10H_, S_5T10H_ and S_10T10H_, over the storage time at 70 °C, up to a very advanced oxidation stage, of: (**a**) mHO-c(*Z*,*E*)-dEs; (**b**) *non-vicinal* mHO-mEPO-*E*-mEs.

It can be observed in Figure 5 that the formation onset of both types of oxylipins occurs at almost the same storage time in the same sample, with the same temporal differences between samples with different types of enrichment. This shows that the different types of sunflower oil enrichment affect the formation of both types of compounds in the same way. Moreover, as shown in Figure 4 and Figure 5, the initiation of the formation of both kinds of compounds is delayed with respect to that of mHPO-c(*E*,*E*)-dEs by about 2–3 days in samples S, S_5T_ and S_10T_, about 3–4 days in samples S_5T10H_ and S_10T10H_, and about 6 days in sample S_10H_. Despite this time lag in the onset of formation, the evolution of their concentration in each sample could be considered, to a certain extent, as parallel to that of mHPO-c(*E*,*E*)-dEs, although both reach much lower values than these latter oxylipins. Finally, it only remains to add that, as shown in Figure 5, both types of oxylipins, like their precursor, are intermediate oxidation compounds.

(D) *Evolution of the concentration of oxylipins bearing hydroxy group.* In this subgroup, monohydroxy-conjugated *Z*,*E*-dienes (mHO-c(*Z*,*E*)-dEs) and *non-vicinal* monohydroxy monoepoxy *E*-monoenes (*non-vicinal* mHO-mEPO-*E*-mEs) are included. The formation of this type of oxylipins is known to occur in the oxidation process of edible oils subjected to accelerated storage conditions [25,26,27,28,29,30,31,52] and they are considered to be derived from mHPO-c(*Z*,*E*)-dEs [82,85,99]. Figure 6 shows the evolution of the concentration of these oxylipins in the different samples over the accelerated storage time, whose differences can only be attributed to the enrichment of the oil in each case.

The formation of mHO-c(*Z*,*E*)-dEs in the samples enriched in αT starts earlier than in the unenriched sample S, while the opposite occurs in the sample enriched in HTy-Ac as shown in Figure 6a, in agreement with that observed in the formation of mHPO-c(*Z*,*E*)-dEs. Their concentration increases until it reaches the maximum value. This is very low in sample S_5T_ and somewhat higher in samples S_10T_ and S_10H_. After that, the concentration starts to decrease in the samples enriched in αT which coincides with the depletion of αT. However, in the S_10H_ sample, the concentration of mHO-c(*Z*,*E*)-dEs does not decrease until almost after 28–30 days of storage, coinciding with the total depletion of HTy-Ac. In the samples enriched in binary mixtures of αT and HTy-Ac, the interactions between both compounds influence the formation of these oxylipins, similar, to some extent, to that observed in the formation of mHPO-c(*Z*,*E*)-dEs. In the first stage, the effect of αT predominates over that of HTy-Ac, so that the formation of these oxylipins occurs earlier in sample S_10T10H_ than in sample S, and only slightly later in sample S_5T10H_. In both samples S_5T10H_ and S_10T10H_, mHO-c(*Z*,*E*)-dEs reach a higher concentration than in the others, and the maximum is reached after 13–14 days of storage, after which it begins to decrease, coinciding with total HTy-Ac depletion.

**Figure 7:**
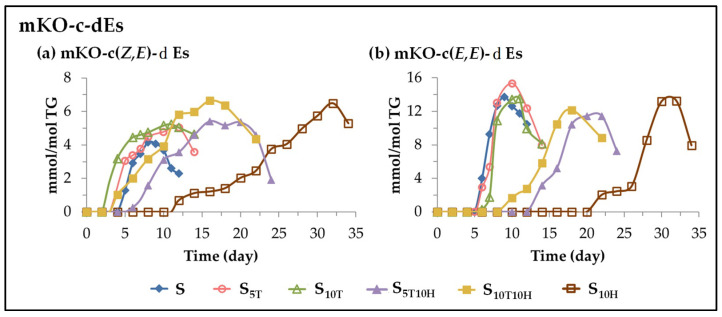
Evolution of the concentration, expressed in mmol per mol of triglyceride (mmol/mol TG), in samples S, S_5T_, S_10T_, S_10H_, S_5T10H_ and S_10T10H_, over the storage time at 70 °C, up to a very advanced oxidation stage, of: (**a**) mKO-c(*Z*,*E*)-dEs; (**b**) mKO-c(*E*,*E*)-dEs.

However, the evolution of the concentration of *non-vicinal* mHO-mEPO-*E*-mEs in the different samples over the storage time, as shown in Figure 6b, is quite different from that of its precursor mHPO-c(*Z*,*E*)-dEs [82,85]. The effect of the different sunflower oil enrichments mentioned above on the evolution of the concentration of these oxylipins is similar, to some extent, to that observed in the evolution of the concentration of the *non-vicinal* mHPO-mEPO-*E*-mEs discussed above. Although the onset of mHO-mEPO-*E*-mEs formation occurs later than that of mHPO-mEPO-*E*-mEs, the storage time at which both oxylipins reach their maximum concentration coincides, the latter being much higher than the former.

(E) *Evolution of the concentration of monoketo-conjugated dienes (mKO-c-dEs)*. This subgroup includes monoketo-conjugated *Z*,*E*-dienes (mKO-c(*Z*,*E*)-dEs) and monoketo-conjugated *E*,*E*-dienes (mKO-c(*E*,*E*)-dEs). These oxylipins are also known to be derived from mHPO-c(*Z*,*E*)-dEs [82,85,99]. The evolution of their concentration over the storage time is represented in Figure 7 from which the effect of the different kinds of oil enrichment on the formation of these oxylipins can be elucidated.

The evolution of the concentration of mKO-c(*Z*,*E*)-dEs has some similarity with that of mHO-c(*Z*,*E*)-dEs, as shown in Figure 6a and Figure 7a. In fact, some authors have described that the latter can be precursors of the former [100]. In the samples enriched in αT, the onset of the formation of these oxylipins is earlier than in the S sample and their rate of formation is greater the higher the enrichment degree in αT. However, in the sample enriched in HTy-Ac, the onset of the formation of these oxylipins is later than in the S, S_5T_ and S_10T_ samples and they reach the maximum concentration after approximately 32 days of storage. In the oil samples enriched in the binary mixtures, the formation of these oxylipins is affected by the interactions between αT and HTy-Ac. It is due to them that the formation of these oxylipins occurs earlier in sample S_10T10H_ than in sample S, and only slightly later in sample S_5T10H_. The maximum concentration is reached after 15 days of storage, after which it begins to decrease, coinciding with the total depletion of HTy-Ac.

**Figure 8:**
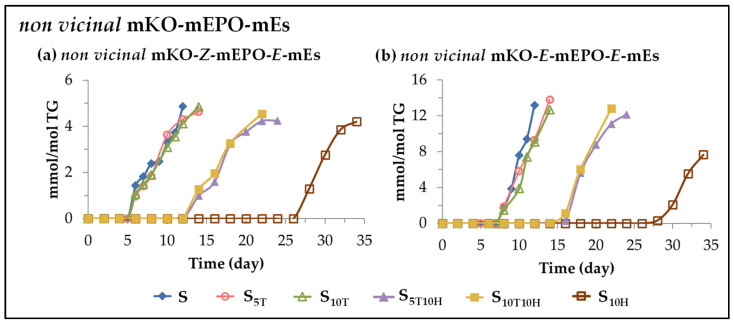
Evolution of the concentration, expressed in mmol per mol of triglyceride (mmol/mol TG), in samples S, S_5T_, S_10T_, S_10H_, S_5T10H_ and S_10T10H_ over the storage time at 70 °C, up to a very advanced oxidation stage, of: (**a**) *non-vicinal* mKO-*Z*-mEPO-*E*-mEs; (**b**) *non-vicinal* mKO-*E*-mEPO-*E*-mEs.

The evolution of the concentration of mKO-c(*E*,*E*)-dEs in the different samples over the storage time also shows the existence of three path groups, namely, the one including samples S, S_5T_ and S_10T_, the one including the sample enriched exclusively in HTy-Ac, S_10H_, and the one corresponding to samples S_5T10H_ and S_10T10H_ in which the effect of the interactions between αT and HTy-Ac is evidenced. Furthermore, the evolution of the concentration of these oxylipins in all the samples resembles that of mHPO-c(*E*,*E*)-dEs, dHPO-nc(*E*,*E*)-dEs, *non-vicinal* mHPO-mEPO-mEs and *non-vicinal* mHO-mEPO-*E*-mEs, as shown in Figure 4, Figure 5, Figure 6b and Figure 7b.

(F) *Evolution of the concentration of other long chain oxylipins bearing keto group*. This subgroup includes *non-vicinal* monoketo *Z*-monoepoxy *E*-monoenes (*non-vicinal* mKO-*Z*-mEPO-*E*-mEs), and *non-vicinal* monoketo *E*-monoepoxy *E*-monoenes (*non-vicinal* mKO-*E*-mEPO-*E*-mEs). The formation of both types of compounds has recently been reported in the oxidation processes of edible oils subjected to accelerated storage [28,29,30,31] and they derive from mHPO-c(*Z*,*E*)-dEs [82,83,85,100].These oxylipins are also formed during the accelerated storage of the sunflower oil samples studied here. The evolution of their concentration in the different samples over the storage time is shown in Figure 8.

The formation of both types of compounds is clearly differentiated in the three groups mentioned above, again showing the individual effects of αT, HTy-Ac and their mixtures in the course of sunflower oil oxidation and also in the formation of both kinds of oxylipins.

Their formation, in samples S_5T_ and S_10T_ occurs when αT is totally depleted or its concentration is very low, in samples S_5T10H_, S_10T10H_ and S_10H_ when HTy-Ac is totally depleted or its concentration is very low, and in all cases when the concentration of their precursor, mHPO-c(*Z*,*E*)-dEs, is diminishing. Nevertheless, the formation of *non-vicinal* mKO-*Z*-mEPO-*E*-mEs occurs somewhat earlier than that of *non-vicinal* mKO-*E*-mEPO-*E*-mEs, probably due to their different isomerism. It is noteworthy that, as Figure 8 indicates, these compounds in all samples can be considered end oxidation products.

#### 3.3.2. Oxylipins Originating from the Cleavage of Long Chain Oxylipins

**Figure 9:**
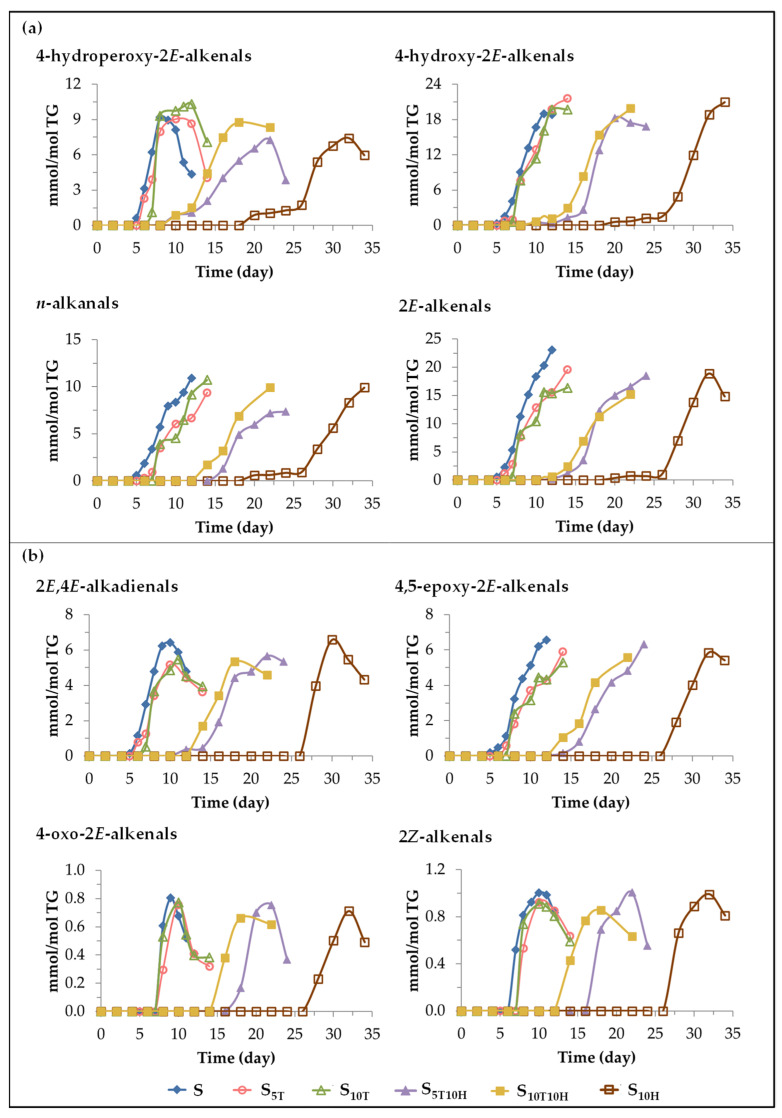
Evolution of the concentration, expressed in mmol per mol of triglyceride (mmol/mol TG), in samples S, S_5T_, S_10T_, S_10H_, S_5T10H_ and S_10T10H_, over the storage time at 70 °C, up to a very advanced oxidation stage, of: (**a**) 4-hydroperoxy-2*E*-alkenals, 4-hydroxy-2*E*-alkenals, *n*-alkanals and 2*E*-alkenals; (**b**) 2*E*,4*E*-alkadienals, 4,5-epoxy-2*E*-alkenals, 4-oxo-2*E*-alkenals and 2*Z*-alkenals.

The number of oxylipins formed by the cleavage of long chain oxidation compounds is very large and a considerable number of them are detectable by ^1^H NMR, as has been demonstrated in previous studies on the oxidation of different edible oils under very varied conditions [25,26,27,29,30,31,45,50,51,101,102,103,104]. They can be small molecules or they can be truncated acyl groups. Most of those detected here contain the aldehyde group and furthermore, in some cases, they also contain a second oxygenated functional group. Their formation and the evolution of their concentration, as could not be otherwise, is also affected by the enrichment of the oil in αT and HTy-Ac or in binary mixtures of them. According to their functional groups, they have been grouped into two subgroups, which will be discussed below.

(A) *Evolution of the concentration of oxylipins bearing aldehyde functional group*. These oxylipins are considered to be derived directly from mHPO-c(*Z*,*E*)-dEs or from intermediate compounds derived from the latter [92,94,95,98,105]. It is also known that some of them are in turn precursors of other oxylipins of this same subgroup. This is the case of4-hydroperoxy-2*E*-alkenals that have been described as precursors of 4-hydroxy-2*E*-alkenals and 4-oxo-2*E*-alkenals [98,106,107]. Likewise, it has also been described that 2*E*,4*E*-alkenals are precursors of 4,5-epoxy-2*E*-alkenals [97,108]. The evolution of the concentration of each of those found in the different samples throughout the storage time is represented in Figure 9, and the effect of oil enrichment on their formation will be discussed below.

(*i*) *Effect caused by the enrichment in αT.* The onset of formation of these oxylipins in samples S, S_5T_ and S_10T_ is very close, as shown in Figure 9, although it occurs slightly later and, in general, their concentration increases at a somewhat slower rate in the samples enriched in αT than in sample S. Consequently, in general, the maximum attained concentration of these oxylipins is also somewhat lower in the samples enriched in αT than in S, except in the case of 4-hydroperoxy-2*E*-alkenals and 4-hydroxy-2*E*-alkenals. This fact is important because the latter oxylipins are considered to be responsible for different degenerative diseases [78,102,109,110,111].

(*ii*) *The effect caused by the enrichment in HTy-Ac*. Two different paths can be distinguished regarding the formation and evolution of the concentration of these oxylipins in the sample enriched in HTy-Ac. In one of them (see Figure 9a), the formation onset of some of these oxylipins occurs after 18–20 days of storage, when the concentration of mHPO-c(*Z*,*E*)-dEs is still in the maximum, and over a long period of time their concentration increases very slowly until the concentration of HTy-Ac is very low, close to its total depletion, after which the concentration of these oxylipins increases very rapidly. In the other path (see Figure 9b), the formation onset of some of these oxylipins occurs later, after 26 days of storage, coinciding in time with a very low concentration of HTy-Ac, and with the start of the decrease of the concentration of mHPO-c(*Z*,*E*)-dEs. The maximum concentration reached by all these oxylipins in sample S_10H_ is very similar to that reached in samples S, S_5T_ and S_10T_ as Figure 9 shows.

(*iii*) *Effect caused by the enrichment in binary mixtures of αT and HTy-Ac*. The most important differences found in the formation of aldehydes between the samples enriched in αT and in HTy-Ac are those related to the onset of their formation. Therefore, it is to be expected that the effect of the interactions between αT and HTy-Ac in the samples enriched in the binary mixtures of these compounds will also be observed in the onset of the formation of these oxylipins. As Figure 9 shows, the onset of aldehydes formation in the S_5T10H_ and S_10T10H_ samples is delayed with respect to that observed in the samples enriched in αT, with this effect being more noticeable the higher the ratio between the concentrations of HTy-Ac and αT. However, this onset is advanced with respect to that observed in the sample enriched in HTy-Ac, this effect being more noticeable the higher the ratio between the concentrations of αT and HTy-Ac. These same effects have been observed in the formation of some long chain oxylipins which originate in the peroxidation of linoleic group, such as mHPO-c(*E*,*E*)dEs, dHPO-nc(*E*,*E*)-dEs, *non-vicinal* mHPO-mEPO-*E*-mEs, *non-vicinal* mHO-mEPO-*E*-mEs, *non-vicinal* mKO-*Z*-mEPO-*E*-mEs, and *non-vicinal* mKO-*E*-mEPO-*E*-mEs, some of which have been previously described as precursors of oxylipins bearing an aldehyde group [71,93,94,95,96,97,98,105] as mentioned above. This suggests that the temporal milestones occurred in the formation of these long chain oxylipins by the effect of binary mixtures allows one to anticipate the effect on the formation of those bearing an aldehyde group.

**Figure 10:**
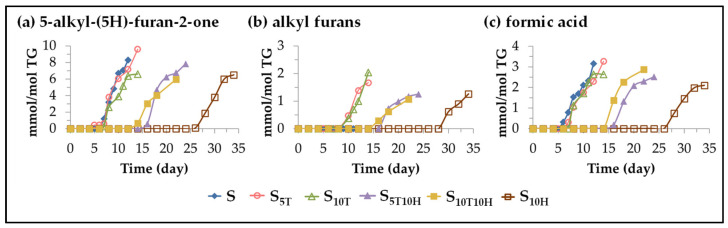
Evolution of the concentration, expressed in mmol per mol of triglyceride (mmol/mol TG), in samples S, S_5T_, S_10T_, S_10H_, S_5T10H_ and S_10T10H_, over the storage time at 70 °C, up to a very advanced oxidation stage, of: (**a**) 5-alkyl-(5H)-furan-2-one; (**b**) alkyl furans; (**c**) formic acid.

It only remains to add that, as Figure 9 clearly shows, some aldehydes, such as 4-hydroperoxy-2*E*-alkenals and 2*E*,4*E*-alkenals are intermediate oxidation compounds.

(B) *Evolution of the concentration of oxylipins not bearing the aldehyde functional group*. This subgroup includes oxidation compounds with very different functional groups, such as 5-alkyl-(5H)-furan-2-ones, 5-alkyl-furans and formic acid. The formation of these three types of oxylipins in the oxidation processes of edible oils has been demonstrated in previous studies both by solid-phase microextraction followed by gas chromatography-mass spectrometry [47,112,113,114], and by ^1^H NMR spectroscopy [29,30,31]. Figure 10 shows the evolution of the concentration of each of these oxylipins in the different samples over the storage time.

This figure shows that the onset of their formation follows the same patterns observed in the onset of the formation of many of the oxylipins mentioned above. This takes place around three storage times (days: 7–8; 14–16; and 26–28) in the three groups of samples (S, S_5T_ and S_10T_; S_5T10H_ and S_10T10H_; and S_10H_). In sunflower oil samples enriched in binary mixtures, the onset of the formation of all these oxylipins occurs at an intermediate storage time, compared to that observed in sunflower oil samples enriched in αT or HTy-Ac, due to the interactions between them discussed above.

#### 3.3.3. Long Chain Oxylipins with Origin in Epoxidation of the Linoleic Acyl Group

The formation of epoxy groups on the unmodified linoleic acyl group has been reported in previous studies on lipid oxidation and several formation mechanisms have been described as possible [67,115,116,117,118,119,120,121]. In fact, the presence of long chains bearing monoepoxy monoene groups in edible oils subjected to accelerated storage conditions, among which the well-known leucotoxin and isoleucotoxin structures can be found, has been proved [26,27,29,30,31].

**Figure 11:**
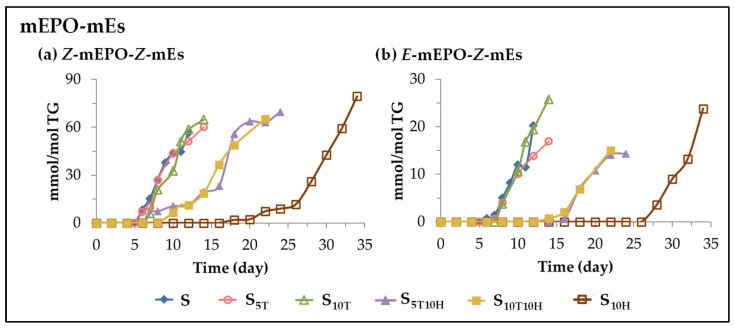
Evolution of the concentration, expressed in mmol per mol of triglyceride (mmol/mol TG), in samples S, S_5T_, S_10T_, S_10H_, S_5T10H_ and S_10T10H_, over the storage time at 70 °C, up to a very advanced oxidation stage, of: (**a**) *Z*-mEPO-*Z*-mEs; (**b**) *E*-mEPO-*Z*-mEs.

Furthermore, the opening of the oxirane ring of the aforementioned monoepoxy monoene long chains provides different pathways, leading to the formation of other oxylipins, some of which incorporate branches to the long chains. This is possible because in the oxidation process of the sunflower oil under accelerated storage conditions, a large number of acids and primary and secondary alcohols are formed [112,113,122] that are capable of opening the oxirane ring by hydrolysis [60,123,124,125]. Due to this, the formation of oxylipins bearing *vicinal* dihydroxy groups can be produced, including the well-known leukotoxin and isoleukotoxin diols [117,123,124,126,127,128,129,130,131]. Likewise, the opening of the oxirane ring caused by acids leads to the formation of ester groups [124,126,127,128,129,132]. In fact, in edible oils submitted to accelerated storage, the formation of formate groups has been proved, which may be either forming *vicinal* diformate structures or *vicinal* monoformate monohydroxy structures [29,30,31,126]. Finally, when the oxirane ring opening is caused by primary or secondary alcohols, ether groups are formed, giving rise to *vicinal* monoether monohydroxy structures [29,30,31,124,127,130,131,133,134,135]. As mentioned above, the formation of the latter two types of oxylipins bearing ester or ether groups involves the introduction of branching in the long chains of the acyl groups, which leads to an increase in the viscosity of the oil and even to its polymerization when the reactions occur between acyl groups. Some of these oxylipins could also be formed endogenously after the action of cytochrome P450 epoxygenases [136,137]. The effect of the enrichment of sunflower oil in αT, in HTy-Ac and in their binary mixtures on the formation and evolution of the concentration of the aforementioned oxylipins will be discussed below.

(A) *Evolution of the concentration of long chains bearing monoepoxy monoene groups*. This subgroup includes two types of long chains bearing monoepoxy monoene groups (mEPO-mE) detected in sunflower oil and other edible oils subjected to accelerated storage conditions [25,26,27,29,30,31]. They are differentiated by their isomerism, some of them exhibit *Z*-monoepoxy-*Z*-monoene groups (*Z*-mEPO-*Z*-mEs) and others *E*-monoepoxy-*Z*-monoene groups (*E*-mEPO-*Z*-mEs) [29,30,31,117]. Figure 11 shows the evolution of their concentration in the different sunflower oil samples throughout the storage time.

It can be observed in this figure that the storage time at which the formation of the long chains bearing *Z*-monoepoxy-*Z*-monoene groups begins is similar to that of the 4-hydroperoxy-2*E*-alkenals in the different samples, and there is also a certain parallelism in the evolution of their concentration, although the former reach a much higher concentration than the latter, which is only slightly lower than that reached by mHPO-c(*E*,*E*)-dEs in all samples, as Figure 4 and Figure 11a show.

**Figure 12:**
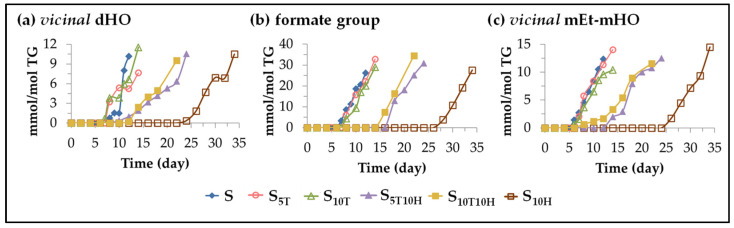
Evolution of the concentration, expressed in mmol per mol of triglyceride (mmol/mol TG), in samples S, S_5T_, S_10T_, S_10H_, S_5T10H_ and S_10T10H_, over the storage time at 70 °C, up to a very advanced oxidation stage, of: (**a**) *vicinal* dHO; (**b**) formate group; (**c**) *vicinal* mEt-mHO.

The formation of long chains bearing *E*-monoepoxy-*Z*-monoene groups, as shown in Figure 11b, starts somewhat later than that of their homologous isomers mentioned above and coincides in time with that of oxylipins that originate from the cleavage of long chains but do not bear aldehyde groups (see Figure 10). The concentration reached by these oxylipins, although much lower than that of their isomers, is also important, and the effect that the enrichment of the oil in αT, in HTy-Ac, or in binary mixtures of them, produces on their formation, is the same as explained above in the formation of some oxylipins such as 5-alkyl-(5H)-furan-2-ones or formic acid.

(B) *Evolution of the concentration of oxylipins with origin in the oxirane ring opening*. As mentioned previously, this subgroup includes long chains bearing *vicinal* dihydroxy monoene groups (*vicinal* dHO-mEs), *vicinal* diformate monoene groups (*vicinal* dF-mEs), *vicinal* monoformate monohydroxy monoene groups (*vicinal* mF-mHO-mEs) or *vicinal* monoether monohydroxy monoene groups (*vicinal* mEt-mHO-mEs). The evolution of the concentration in the different samples over the storage time of some of the aforementioned groups such as *vicinal* dHO, formate and *vicinal* mEt-mHO groups, is shown in Figure 12.

The effect of sunflower oil enrichment in HTy-Ac, in αT and in binary mixtures of HTy-Ac and αT on the onset of the formation of long chains with *vicinal* dihydroxy groups (*vicinal* dHO) or with *vicinal* monoether monohydroxy groups (*vicinal* mEt-mHO) is similar, to some extent to that observed in the onset of the formation of 4-hydroxy-2*E*-alkenals, as shown in Figure 9a and Figure 12. However, the effect of different types of oil enrichment on the onset of formate group formation is very similar to that observed on the onset of formation of its precursor, formic acid, and on the onset of the formation of long chains with *E*-monoepoxy-*Z*-monoene groups, which could also be precursors of formate groups (see Figure 10, Figure 11b and Figure 12). The concentration reached by formate groups can be considered remarkable, which indicates the relevance of the formation of ester groups during oil oxidation under accelerated conditions and, as a consequence, the generation of branchings in the long chain acyl groups with repercussions on oil viscosity or even its polymerization.

### 3.4. Lipolysis Extent and 1,2-diglycerides Formation in the Different Sunflower Samples throughout the Accelerated Storage Time: Influence of the Enrichment in αT, HTy-Ac and in Binary Mixtures of These

**Figure 13:**
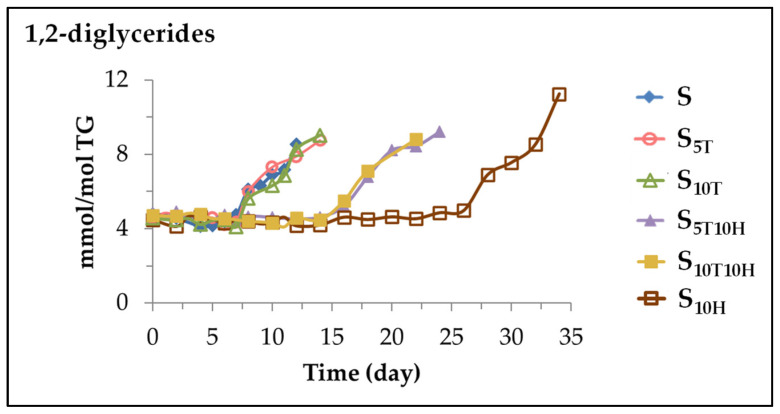
Evolution of the concentration of 1,2-diglycerides, expressed in mmol per mol of triglyceride (mmol/mol TG), in samples S, S_5T_, S_10T_, S_10H_, S_5T10H_ and S_10T10H_, over the storage time at 70 °C, up to a very advanced oxidation stage.

This sunflower oil has a very small concentration of 1,2-diglycerides as usual. When this oil is subjected to accelerated storage, lipolysis occurs to a very low degree, producing 1,2-diglycerides [31]. Figure 13 shows the evolution of the concentration of these glycerides in the different oil samples.

It can be observed in Figure 13 that the storage time at which these glycerides are formed is also affected by the type of oil enrichment. Thus, in samples S, S_5T_ and S_10T_ the formation of 1,2-diglycerides begins almost at the same time of storage in the three samples, although in the samples enriched in αT the concentration reached is higher than in sample S. In the sample enriched in HTy-Ac the formation of this glyceride starts much later than in the samples mentioned above, and it occurs when the HTy-Ac is almost completely depleted. In this sample, 1,2-diglycerides reach a higher concentration than in the other samples. Finally, in samples S_5T10H_ and S_10T10H_, as a consequence of the interactions between αT and HTy-Ac, the formation of these glycerides occurs earlier than in S_10H_, but also when HTy-Ac is totally depleted and the concentration they reach is only slightly higher than that reached in samples S_5T_ and S_10T_. It is evident that, in sunflower oil subjected to accelerated storage conditions, not only does oxidation occur, but so does hydrolysis. Although the latter reaction occurs to a very small extent, it is affected by the enrichment of the oil in αT, in Hty-Ac or in their binary mixtures, as has been shown.

## 4. Conclusions

The methodology based on ^1^H NMR spectroscopy used here to study the individual and joint effect of *alpha*-tocopherol, αT, and hydroxytyrosol acetate, HTy-Ac, on the evolution of an edible oil rich in omega-6 acyl groups subjected to oxidative conditions, such as accelerated storage, provides simultaneously a wealth of information impossible to obtain by any other method to date, and without subjecting the sample to chemical modifications or prior separation steps. This information concerns not only the degradation of the main component of the oil and the added αT and HTy-Ac, but also the formation and evolution of the concentration of a large number of oxylipins, as well as the occurrence of some lipolysis during the accelerated storage of the oil.

The main component of the oil, that is to say the linoleic acyl group, degrades during accelerated storage, and two stages with different degradation rates can be distinguished in its degradation path. Taking the degradation process of the non-enriched sample as a reference, the enrichment of the oil in αT accelerates the degradation of the linoleic acyl group in the first stage of this process and slows down its degradation in the second stage, whereas the enrichment of the oil in HTy-Ac slows down the degradation of the linoleic acyl group in both stages of the process. It follows that enrichment of the oil in αT leads to a prooxidant effect in the first stage of the oxidation process, but an antioxidant effect in the second stage, whereas enrichment of the oil in HTy-Ac causes an antioxidant effect in both stages of the oxidation process. The joint enrichment of the oil in αT and HTy-Ac accelerates the degradation of the linoleic acyl group in the first stage, compared to that observed in the non-enriched S sample, although to a lesser extent than that caused by the enrichment in αT, and slows down the degradation in the second stage to a greater extent than that produced by the enrichment with either of these compounds individually, so that the duration of this second stage in the samples enriched in binary mixtures is the longest of all. It follows that the enrichment of the oil in both binary mixtures leads to a prooxidant effect in the first stage of oil degradation, and an antioxidant effect in the second stage, and a total duration of the oxidation process intermediate between that of the oil enriched individually in αT and in HTy-Ac.

Under the aforementioned oxidative conditions, αT and HTy-Ac are also degraded, the former being fully depleted much earlier than the latter. The storage time at which the total depletion of these compounds occurs is very important in the evolution of the oil oxidation process. Thus, the end of the first stage of the degradation of the linoleic group and the beginning of the second stage coincides with the storage time at which the total depletion of αT occurs in the samples enriched in this compound, or with the storage time at which the total depletion of HTy-Ac occurs in the samples enriched in HTy-Ac, and also in the binary mixtures of αT and HTy-Ac. It should be highlighted that, consequently, the second stage of degradation of the linoleic acyl groups in all samples takes place in the absence of αT and HTy-Ac, however, it can be said that the evolution of the oil at this stage is also affected by the initial enrichment in them. It is also very noteworthy that in both samples enriched in binary mixtures the total depletion of αT occurs later than in the samples enriched exclusively in this compound, while the opposite happens to HTy-Ac. Both facts are due to the interactions between αT and HTy-Ac which determine the evolution of oil oxidation in the samples enriched in binary mixtures.

Among the oxylipins formed, a large number originate from the peroxidation of the main oil component; another large group originates from the cleavage of long-chain oxylipins; and finally, another group originates from the epoxidation of the main component of the oil. The latter, in turn, after opening their oxirane ring with acids or alcohols, give rise to others that have branches in the chains or may even cause polymerization of the oil.

The first oxylipins formed carry monohydroperoxy-conjugated dienes, mHPO-c-dEs, and can be either mHPO-c(*Z*,*E*)-dEs or mHPO-c(*E*,*E*)-dEs. In sample S, the formation and degradation of both types of oxylipins runs in parallel, the latter always in a higher concentration than the former.

Enrichment in αT advances and accelerates the formation of mHPO-c(*Z*,*E*)-dEs, but delays and slows down the formation of mHPO-c(*E*,*E*)-dEs in relation to what occurs in S. Therefore, the concentration reached by the former compounds in the samples enriched in αT is higher than in the S sample, and that reached by the latter is lower to a greater extent the higher the level of enrichment. This is in line with the aforementioned dual effect of αT enrichment, a prooxidant effect in the first stage of oil degradation and an antioxidant effect in the second stage of oil degradation.

The enrichment of the oil in HTy-Ac delays and slows down the formation of both mHPO-c-dEs in relation to what occurs in the S sample, and as a consequence, the maximum concentration reached by them is lower than in the S sample and is also reached much later. Furthermore, this enrichment leads to large differences in the evolution of the concentration of mHPO-c(*Z*,*E*)-dEs and mHPO-c(*E*,*E*)-dEs. Thus, mHPO-c(*Z*,*E*)-dEs reach their highest concentration when HTy-Ac exhibits the highest degradation rate, and this concentration remains stabilized for a long period of time, until the total depletion of HTy-Ac, after which it decreases, coinciding with the sudden increase in the concentration of mHPO-c(*E*,*E*)-dEs and with the beginning of the second stage of linoleic degradation.

The enrichment of the oil in binary mixtures of αT and HTy-Ac advances and accelerates the formation of mHPO-c(*Z*,*E*)-dEs to a lesser extent than in the samples enriched in αT, but to a greater extent than in the S sample, and their concentration increases during a longer period of time than in the samples enriched in αT, achieving a higher concentration than in these latter samples. This is because in the samples enriched in binary mixtures the total depletion of αT occurs later than in the oil samples enriched only in αT, which could be attributed to the fact that HTy-Ac regenerates it to some extent and, therefore, HTy-Ac degrades earlier in these samples than in the sample enriched only in HTy-Ac. These facts determine not only the concentration of mHPO-c(*Z*,*E*)-dEs, but also their subsequent evolution and that of mHPO-c(*E*,*E*)-dEs, as well as the main milestones of the total oxidation process. Among them, in addition to the abovementioned, the duration of the stabilization of mHPO-c(*Z*,*E*)-dEs concentration, the beginning of their degradation, the total depletion of HTy-Ac, the change in the rate of linoleic acyl group degradation, the sudden increase in mHPO-c(*E*,*E*)-dEs concentration, the time at which these latter oxylipins reach their maximum concentration, and the total duration of the oxidation process, as well as the formation and evolution of the concentration of the other oxylipins and even the occurrence of certain lipolysis can be cited.

It only remains to be added that the formation and evolution of the concentration of the other oxylipins mentioned above are also governed by αT or HTy-Ac in samples enriched in these compounds and by interactions between them in samples enriched in binary mixtures. To the best of our knowledge, this is the first time that the above indicated milestones concerning the main aspects involved in the oxidation process of real systems, such as those studied here, have been shown to fit together like the pieces of a puzzle.

## Figures and Tables

**Table 1:** Degradation rates (D_R1L_ and D_R2L_), expressed in mmol of linoleic acyl group per mol of triglyceride and day (mmol mol^−1^ TG day^−1^), in the two linear stages of its degradation path depicted in Figure 1, of each one of the samples, S, S_5T_, S_10T_, S_10H_, S_5T10H_ and S_10T10H_, over the storage time at 70 °C, which coincide with the slopes of these linear stages. The correlation coefficients of the corresponding equations are given in brackets.

Samples	First Stage			Second Stage	
	Time (Days)	D_R1L_ (mmol/mol TG Day)		Time (Days)	D_R2L_ (mmol/mol TG Day)
**S**	0–5	−23.5 (0.95)		5–12	−185.8 (0.97)
**S_5T_**	0–5	−44.1 (1.00)		5–14	−137.2 (0.95)
**S_10T_**	0–7	−61.7 (0.96)		7–14	−133.5 (0.95)
**S_10H_**	0–26	−12.0 (0.99)		26–34	−140.8 (0.99)
**S_5T10H_**	0–14	−29.4 (0.98)		14–24	−110.8 (0.91)
**S_10T10H_**	0–12	−38.9 (0.99)		12–24	−102.8 (0.99)

**Table 2:** Degradation rates of *alpha*-tocopherol (D_RT_) and of hydroxytyrosol acetate (D_RH_) expressed in mmol mol^−1^ TG day^−1^ in the linear stages of its degradation path, depicted in Figure 2, in each one of the samples, S_5T_, S_10T_, S_10H_, S_5T10H_ and S_10T10H_ over the storage time at 70 °C. The correlation coefficients of these lines are given in brackets.

Samples	Degradation Stage	*Alpha*-Tocopherol (αT)			Hydroxytyrosol Acetate (HTy-Ac)	
		Time (Days)	D_RT_ (mmol/mol TG Day)		Time (Days)	D_RH_ (mmol/mol TG Day)
**S_5T_**	-	0–5	1.05 (0.99)		-	-
**S_10T_**	-	0–7	1.30 (0.99)		-	-
**S_10H_**	1st	-	-		0–12	0.20 (0.95)
	2nd	-	-		12–28	0.52 (0.99)
**S_5T10H_**	1st	0–8	0.58 (0.96)		0–7	0.54 (0.98)
	2nd	-	-		7–14	0.92 (0.98)
**S_10T10H_**	1st	0–10	0.99 (0.99)		0–10	0.56 (0.97)
	2nd	-	-		10–14	1.11 (0.94)

## Data Availability

Data is contained within the article and supplementary material.

## Supplementary Materials

The following supporting information can be downloaded at: https://www.mdpi.com/article/10.3390/antiox11061156/s1, Table S1. Standard compounds used for identification purposes; Table S2. ^1^H NMR signals obtained in CDCl_3_ of protons of main sunflower oil components, their chemical shifts, multiplicities and assignments to protons of different functional groups present in edible oils; Table S3. Chemical shift assignments and multiplicities of ^1^H NMR signals in CDCl_3_ of protons of hydroxytyrosol acetate and *alpha*-tocopherol; Table S4. Chemical shift assignments and multiplicities of ^1^H NMR signals in CDCl_3_ of protons of monohydroperoxy-conjugated octadecadienes (mHPO-c-dEs) derived from linoleic groups; Table S5. Chemical shift assignments and multiplicities of ^1^H NMR signals in CDCl_3_ of protons of dihydroperoxy non-conjugated *E*,*E*-octadecadienes (dHPO-nc(*E*,*E*)-dEs); Table S6. Chemical shift assignments and multiplicities of ^1^H NMR signals in CDCl_3_ of protons of *non vicinal* monohydroperoxy monoepoxy *E*-octadecamonoenes (mHPO-mEPO-*E*-mEs); Table S7. Chemical shift assignments and multiplicities of ^1^H NMR signals in CDCl_3_ of protons of monohydroxy-conjugated *Z*,*E*-octadecadienes (mHO-c(*Z*,*E*)-dEs); Table S8. Chemical shift assignments and multiplicities of ^1^H NMR signals in CDCl_3_ of protons of monohydroxy monoepoxy *E*-octadecamonoenes (mHO-mEPO-*E*-mEs); Table S9. Chemical shift assignments and multiplicities of ^1^H NMR signals in CDCl_3_ of protons of monoketo-conjugated octadecadienes (mKO-c-dEs); Table S10. Chemical shift assignments and multiplicities of ^1^H NMR signals in CDCl_3_ of protons of monoketo monoepoxy *E*-octadecamonoenes (mKO-mEPO-*E*-mEs); Table S11. Chemical shift assignments and multiplicities of ^1^H NMR signals in CDCl_3_ of protons of different types of aldehydes; Table S12. Chemical shift assignments and multiplicities of ^1^H NMR signals in CDCl_3_ of protons of furan groups; Table S13. Chemical shift assignments and multiplicities of ^1^H NMR signals in CDCl_3_ of protons of epoxy derivatives derived from linoleic acyl groups; Table S14. Chemical shift assignments and multiplicities of ^1^H NMR signals in CDCl_3_ of protons of different types of dihydroxy groups (dHO); Table S15. Chemical shift assignments and multiplicities of ^1^H NMR signals in CDCl_3_ of protons of formic acid and formates.
