# Supplementary material for: Individual and Joint Effect of Alpha-Tocopherol and Hydroxytyrosol Acetate on the Oxidation of Sunflower Oil Submitted to Oxidative Conditions: A Study by Proton Nuclear Magnetic Resonance
Source: Antioxidants (Basel). 2022 Jun 13;11(6):1156. doi: 10.3390/antiox11061156 (PMC9220198; doi:10.3390/antiox11061156)
Supplement: Supplementary file 1 [file antioxidants-11-01156-s001.zip › antioxidants-1767031-supplementary.pdf]

SUPPLEMENTARY MATERIAL

Individual and Joint Effect of Alpha-Tocopherol and Hydroxytyrosol Acetate on the Oxidation of Sunflower Oil Submitted to Oxidative Conditions: A Study by Proton Nuclear Magnetic Resonance

Sofía del Caño-Ochoa, Ainhoa Ruiz-Aracama, María D Guillén *

Food Technology. Faculty of Pharmacy. Lascaray Research Center. University of the Basque Country (UPV/EHU). Paseo de la Universidad nº 7, 01006 Vitoria, Spain, Telf: 34-945-013081. E-mail: mariadolores.guillen@ehu.eus

Table S1. Standard compounds used for identification purposes.

2*E*-hexenal [a]	2*E*-heptenal [a]
2*E*-decenal [a]	2*E*,4*E*-hexadienal [a]
2*E*,4*E*-heptadienal [a]	2*E*,4*E*-decadienal [a]
4,5-epoxy-2*E*-decenal [a]	12,13-epoxy-9*Z*-octadecenoic acid methyl ester (isoleukotoxin methyl ester) [a]
2-pentylfuran [a]	2-ethylfuran [a]
amylformate [a]	octylfor-mate [a]
alpha-tocopherol [a]	9,10-epoxy-12*Z*-octadecenoic acid (leukotoxin) [b]
12,13-*E*-epoxy-9*Z*-octadecenoic acid [b]	4-hydroxy-2*E*-nonenal [b]
4-hydroperoxy-2*E*-nonenal [b]	4-oxo-2*E*-nonenal [b]
9,10-dihydroxy-12*Z*-octadecenoic acid (leukotoxin diol) [b]	12,13-dihydroxy-9*Z*-octadecenoic acid (isoleukotoxin diol) [b]
9-keto-12,13-*E*-epoxy-10*E*-octadecenoic acid [b]	9-keto-10*E*,12*E*-octadecadienoic acid [b]
9-keto-10*E*,12*Z*-octadecadienoic acid [b]	13-keto-9*Z*,11*E*-octadecadienoic acid [b]
12R-hydroxy-9*Z*-octadecenoic acid methyl ester (ricinoleic acid methyl ester) [b]	9S-hydroxy-10*E*,12*E*-octadecadienoic acid (Dimorphecolic acid) [c]
methyl 13S-hydroxy-9S,10R-epoxy-11*E*-octadecenoate [c]	11S-hydroxy-9S,10S-epoxy-12*Z*-octadecenoic acid methyl ester [c]
13S-hydroxy-11S,12S-epoxy-9*Z*-octadecenoic acid methyl ester [c]	9-hydroxy-10-keto-12*Z*-octadecenoic acid [c]
9,10-*Z*-12,13-*Z*-diepoxyoctadecanoic acid [c]	13-hydroxy-12-keto-9*Z*-octadecenoic acid [c]
hydroxytyrosol acetate [d]	

Acquired from: [a] Sigma-Aldrich (St. Louis, MO, USA); [b] Cayman Chemical (Ann Arbor, MI, USA); [c] Larodan (Malmö, Sweeden); [d] Seprox Biotech (Madrid, Spain).

Table S2. ^1H NMR signals, obtained in $CDCl_3$, of protons of main sunflower oil components, their chemical shifts, multiplicities and assignments to protons of different functional groups present in edible oils.

Chemical shift (ppm) [a, **]	Multiplicity	Functional group	
		Type of protons *	Compound
0.83-0.93	t	-C<u>H</u>3	Saturated, oleic and linoleic acyl group
1.19-1.42	m	-(C<u>H</u>2)n-	Acyl groups
1.52-1.70	m	-OCO-CH2-C<u>H</u>2-	Acyl groups
1.94-2.14	m	-C<u>H</u>2-CH=CH-	Acyl groups
2.23-2.36	dt	-OCO-C<u>H</u>2-	Acyl groups
2.70-2.84	t	=CH-C<u>**H**</u>**2**-CH=	Acyl groups
3.73	m[b]	ROCH2-CH(O*R*')-C<u>**H**</u>**2**OH	1,2-DG
4.10-4.32	dd,dd	-C<u>**H**</u>**2**OCOR	Glyceryl groups
5.20-5.29	m	-C<u>H</u>OCOR	Glyceryl groups
5.29-5.46	m	-C<u>H</u>=C<u>H</u>-	Acyl groups

Abbreviations: dd: double doublet; t: triplet; dt: double triplet; m: multiplet.
*Area of the signals due to the protons in bold were used for the quantification of each compound.
**The assignment of the ^1H NMR signals of the protons was made with the aid of standard compounds.
The assignment of the ^1H NMR signals of the protons of main components was made with the data taken from the literature:
[a] Guillén, M.D.; Ruiz, A. (2003). *Eur. J. Lipid Sci. Tech. 105*(11), 688-696.
[b]This signal shows different multiplicity if the spectrum is acquired from the pure compound or taking part in the mixture.

Table S3. Chemical shift assignments and multiplicities of ^{1}H NMR signals in $CDCl_3$ of protons of *alpha*-tocopherol and hydroxytyrosol acetate.

***Alpha*-tocopherol**

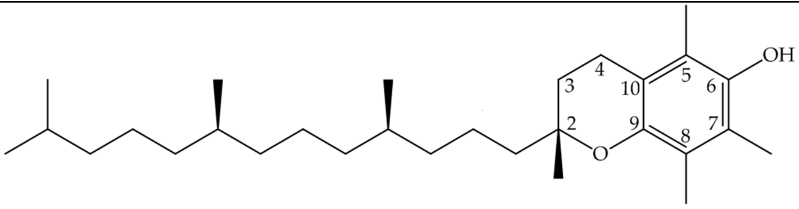

Chemical shift (ppm) [a, **]	Multiplicity	Functional group	Type of protons *
4.18	s	Hydroxy group bonded to C6	-OH
**2.60**	t	Methylene group in position 4	-C**H**$_2$-
2.15	s	Methyl group bonded to C7	-CH_3
2.11	s	Methyl group bonded to C5 and C8	-CH_3
1.8	m	Methylene group in position 3	-CH_2-
1.22	s	Methyl group bonded to C2	-CH_3
1.1-1.5	m	Chain methylene groups<br>Chain methine groups	-CH_2-<br>-CH-
0.83-0.88	m	Chain methyl groups	-CH_3

Hydroxytyrosol acetate

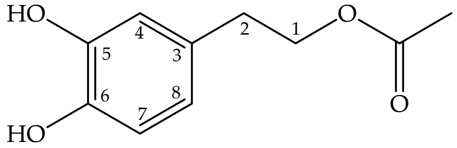

Chemical shift (ppm) [b, **]	Multiplicity	Functional group (Type of protons) *
**6.71**	d	-C**H**=CH- (H-7 of the dihydroxyphenyl group)
6.67	d	=CH- (H-4 of the dihydroxyphenyl group)
6.54	dd	-CH=CH- (H-8 of the dihydroxyphenyl group)
4.12	t	-CH_2-OCO-
2.73	t	-CH_2-CH_2-OCO-
2.00	s	-OCO-CH_3

Abbreviations: s: singlet; d: doublet; dd: double doublet; t: triplet; m: multiplet.

[*] Area of the signals due to the protons in bold, together with the area of the *sn*-1 and *sn*-3 signals of TG shown in Table S1, were used for the quantification of each compound, using the equation [eq. 1] showed in the Materials and Methods.

[**] The assignment of the ^{1}H NMR signals of the protons was made with the aid of standard compounds.

The assignment of the ^{1}H NMR signals of the protons of *alpha*-tocopherol and hydroxytyrosol acetate was made with the data taken from the literature:

[a] Baker, J.K.; Myers, C.W. (1991). *Pharm. Res. 8*(6), 763-770.

[b] Bouallagui, Z.; Bouaziz, M.; Lassoued, S.; Engasser, J.M.; Ghoul, M.; Sayadi, S. (2011). *Appl. Biochem. Biotechnol. 163*(5), 592-599.

[b] Ammendola, S.; Giusti, A. M.; Masci, A.; Mosca, L.; Saso, L.; Bovicelli, P. (2011). *J. Sci. Ind. Res. 70*(11), 929-937.

Table S4. Chemical shift assignments and multiplicities of ^{1}H NMR signals in $CDCl_3$ of protons of monohydroperoxy conjugated octadecadienes (mHPO-c-dEs) coming from linoleic groups.

**mHPO-c(*Z,E*)-dEs** (9-hydroperoxy-10*E*,12*Z*-octadecadienoate; 13-hydroperoxy-9*Z*,11*E*-octadecadienoate)		
Chemical shift (ppm) [a,**]	Multiplicity	Functional group (Type of protons) [*]
**6.58**	dddd	-C**H**=CH-CHOOH-
6.00	ddtd	-CH=CH-CH=CH-CHOOH-
5.56	ddm	-CH=CH-CHOOH-
5.51	dtm	-CH=CH-CH=CH-CHOOH-
4.37	dtd	-CHOOH-
2.30	t	-OCO-CH_2-
2.19	dtd	-CH_2-CH=CH-
1.55-1.71	m	-CHOOH-CH_2-<br>-OCO-CH_2-CH_2-
1.24-1.43	m	-CH_2-
0.89	t	-CH_3
**mHPO-c(*E,E*)-dEs** (9-hydroperoxy-10*E*,12*E*-octadecadienoate; 13-hydroperoxy-9*E*,11*E*-octadecadienoate)		
Chemical shift (ppm) [a,**]	Multiplicity	Functional group (Type of protons) [*]
**6.27**	ddm	-C**H**=CH-CHOOH-
6.06	ddtd	-CH=CH-CH=CH-CHOOH-
5.76	dtm	-CH=CH-CH=CH-CHOOH-
5.47	ddm	-CH=CH-CHOOH-
4.32	dtd	-CHOOH-
2.30	t	-OCO-CH_2 -
2.09	dtd	-CH_2-CH=CH-
1.42-1.66	m	-CHOOH-CH_2-<br>-OCO-CH_2-CH_2 -
1.25-1.43	m	-CH_2-
0.89	t	-CH_3

Abbreviations: dddd: double of double of double doublet; ddtd: double of double of triple doublet; dtd: double of triple doublet; t: triplet; m: multiplet; ddm: double of double multiplet; dtm: double of triple multiplet.

[*]Area of the signals due to the protons in bold, together with the area of the *sn*-1 and *sn*-3 signals of TG shown in Table S1, were used for the quantification of each compound, using the equation [eq. 1] showed in the Materials and Methods.

[**]The assignment of the ^{1}H NMR signals of the protons was made with the aid of standard compounds.

The assignment of the ^{1}H NMR signals of the protons of mHPO-c-dEs derived from linoleic acyl groups was made with the data taken from the literature:

[a] Goicoechea, E.; Guillén, M.D. (2010). *J. Agric. Food Chem. 58*, 6234-6245.

[a] Pajunen, T.I.; Koskela, H.; Hase, T.; Hopia, A. (2008). *Chem. Phys. Lipids. 154,* 105-114.

Table S5. Chemical shift assignments and multiplicities of ^{1}H NMR signals in $CDCl_3$ of protons of dihydroperoxy non conjugated *E,E*-octadecadienes (dHPO-nc(*E,E*)-dEs).

**dHPO-nc(*E,E*)-dEs**<br>(9,12-dihydroperoxy-10*E*,13*E*-octadecadienoate; 10,13-dihydroperoxy-8*E*,11*E*-octadecadienoate)		
Chemical shift (ppm) [a]	Multiplicity	Functional group (Type of protons) *
5.56-5.87	m	-CHOOH-CH=CH-CHOOH-<br>-CHOOH-CH=CH-CH_2-
5.36-5.50	m	-CHOOH-CH=CH-CH_2-
**4.81**	dd	-C**H**OOH-
4.30-4.39	m	-CHOOH-
2.33	t	-OCO-CH_2-
2.02-2.11	m	-CH=CH-CH_2-
1.54-1.69	m	-OCO-CH_2-CH_2-
1.16-1.43	m	-CH_2-
0.81-0.91	t	-CH_3

Abbreviations: dd: double doublet; t: triplet; m: multiplet.

*Area of the signals due to the protons in bold, together with the area of the *sn*-1 and *sn*-3 signals of TG shown in Table S1, were used for the quantification of each compound, using the equation [eq. 1] showed in the Materials and Methods.

The assignment of the ^{1}H NMR signals of the protons of dHPO-nc(*E,E*)-dEs was made with the data taken from the literature:

[a] Zhang, W. (2008). Synthesis and Fragmentation Reactions of Linoleic Acid-Derived Hydroperoxides (Doctoral dissertation, Case Western Reserve University).

Table S6. Chemical shift assignments and multiplicities of ^{1}H NMR signals in $CDCl_3$ of protons of *non vicinal* monohydroperoxy monoepoxy *E*-octadecamonoenes (mHPO-mEPO-*E*-mEs).

mHPO-*E*-mEPO-*E*-mEs

(9-hydroperoxy-12,13-*E*-epoxy-10*E*-octadecenoate)

Chemical shift (ppm) [a]	Multiplicity	Functional group (Type of protons) *
**5.85**	dd	-CHOCH-CH=C**H**-CHOOH-
5.47	dd	-CHOCH-CH=CH-CHOOH-
4.33	m	-CHOOH-
3.11	dd	-CHOCH-CH=CH-
2.84	m	-CHOCH-CH=CH-
2.30	t	-OCO-CH_2-
1.51	m	-OCO-CH_2-CH_2-
1.31	m	-CH_2-
0.89	t	-CH_3

mHPO-*Z*-mEPO-*E*-mEs

(13-hydroperoxy-9,10-*Z*-epoxy-11*E*-octadecenoate)

Chemical shift (ppm) [b]	Multiplicity	Functional group (Type of protons) *
**5.84**	dd	-CHOCH-CH=C**H**-CHOOH-
5.5-5.7	m	-CHOCH-CH=CH-CHOOH-
4.2-4.4	m	-CHOOH-
3.42	dd	-CHOCH-CH=CH-
3.0-3.2	m	-CHOCH-CH=CH-
2.32	t	-OCO-CH_2-
1.1-1.8	m	-CH_2-
0.85	t	-CH_3

Abbreviations: dd: double doublet; t: triplet; m: multiplet.

* Area of the signals due to the protons in bold, together with the area of the *sn*-1 and *sn*-3 signals of TG shown in Table S1, were used for the quantification of each compound, using the equation [eq. 1] showed in the Materials and Methods.

The assignment of the ^{1}H NMR signals of the protons of mHPO-mEPO-*E*-mEs was made with the data taken from the literature:

[a] Gardner, H.W.; Weisleder, D.; Kleiman, R. (1978). *Lipids, 13*(4), 246-252.

[b] Gu, X.; Salomon, R. (2012). *Free Radical Biol. Med. 52*, 601-606.

Table S7. Chemical shift assignments and multiplicities of ^{1}H NMR signals in $CDCl_3$ of protons of monohydroxy conjugated *Z,E*-octadecadienes (mHO-c(*Z,E*)-dEs).

**mHO-c(*Z,E*)-dEs** (13-hydroxy-9*Z*,11*E*-octadecadienoate; 9-hydroxy-10*E*,12*Z*-octadecadienoate)		
Chemical shift (ppm) [a,**]	Multiplicity	Functional group (Type of protons) [*]
**6.46**	ddd	-C**H**=CH-CHOOH-
5.94	dd	-CH=CH-CH=CH-CHOOH-
5.64	dd	-CH=CH-CHOOH-
5.42	ddt	-CH=CH-CH=CH-CHOOH-
4.13	m	-CHOH-
2.27	t	-OCO-CH_2-
2.15	m	-CH=CH-CH_2-
1.2-1.7	m	-CH_2-
0.86	t	-CH_3

Abbreviations: dd: double doublet; ddd: double of double doublet; t: triplet; ddt: double of double triplet; m: multiplet.

[*] Area of the signals due to the protons in bold, together with the area of the *sn*-1 and *sn*-3 signals of TG shown in Table S1, were used for the quantification of each compound, using the equation [eq. 1] showed in the Materials and Methods.

[**] The assignment of the ^{1}H NMR signals of the protons was made with the aid of standard compounds.

The assignment of the ^{1}H NMR signals of the protons of mHO-c(*Z,E*)-dEs was made with the data taken from the literature:

[a] Kuklev, D. V.; Christie, W. W.; Durand, T.; Rossi, J. C.; Vidal, J. P.; Kasyanov, S. P.;... Bezuglov, V. V. (1997). *Chem. Phys. Lipids, 85*(2), 125-134.

Table S8. Chemical shift assignments and multiplicities of ^{1}H NMR signals in $CDCl_3$ of protons of monohydroxy monoepoxy *E*-octadecamonoenes (mHO-mEPO-*E*-mEs).

**mHO-*E*-mEPO-*E*-mEs** (9-hydroxy-12,13-*E*-epoxi-10*E*-octadecenoate; 13-hydroxy-9,10-*E*-epoxy-11*E*-octadecenoate[**])		
Chemical shift (ppm) [a]	Multiplicity	Functional group (Type of protons) [*]
**5.93**	dd	-CH=C**H**-CHOH-
5.41	ddd	-CH=CH-CHOH-
4.14	m	-CHOH-
3.09	dd	-CHOCH-CH=CH-
2.81	dt	-CHOCH-CH=CH-
2.30	t	-OCO-CH_2-
1.49	m	-OCO-CH_2-CH_2-
1.31	m	-CH_2-
0.89	t	-CH_3
**mHO-*Z*-mEPO-*E*-mEs** (13-hydroxy-9,10-Z-epoxy-11*E*-octadecenoate)		
Chemical shift (ppm) [b]	Multiplicity	Functional group (Type of protons) [*]
**5.95**	dd	-CH=C**H**-CHOH-
5.54	ddd	-CH=CH-CHOH-
3.41	dd	-CHOCH-CH=CH-
3.07	dt	-CHOCH-CH=CH-
2.30	t	-OCO-CH_2-
2.10	br	-CHOH-
1.2-1.6	m	-CH_2-
0.89	t	-CH_3

Abbreviations: dd: double doublet; ddd: double of double doublet; t: triplet; dt: double triplet; m: multiplet; br: broad signal.

[*] Area of the signals due to the protons in bold, together with the area of the *sn*-1 and *sn*-3 signals of TG shown in Table S1, were used for the quantification of each compound, using the equation [eq. 1] showed in the Materials and Methods.

[**] The assignment of the ^{1}H NMR signals of the protons was made with the aid of standard compounds.

The assignment of the ^{1}H NMR signals of the protons of mHO-mEPO-*E*-mEs was made with the data taken from the literature:

[a] Gardner, H.W.; Weisleder, D.; Kleiman, R. (1978). *Lipids, 13*(4), 246-252.
[a] Schieberle, P.; Trebert, Y.; Firl, J.; Grosch, W. (1988). *Chem. Phys. Lipids, 48*(3-4), 281-288.
[a] Ramsden, C.E.; Domenichiello, A.F.; Yuan, Z.X.; Sapio, M.R.; Keyes, G.S.; Mishra, S. K.; ... Davis, J.M. (2017). *Sci. Sign. 10*(493), eaal5241.
[b] Hidalgo, F.J.; Zamora, R.; Vioque, E. (1992). *Chem. Phys. Lipids, 60*(3), 225-233.

Table S9. Chemical shift assignments and multiplicities of ^{1}H NMR signals in $CDCl_3$ of protons of monoketo conjugated octadecadienes (mKO-c-dEs).

**mKO-c(*Z,E*)-dEs** (13-keto-9*Z*,11*E*-octadecadienoate; 9-keto-10*E*,12*Z*-octadecadienoate)		
Chemical shift (ppm) [a, **]	Multiplicity	Functional group (Type of protons) [*]
**7.49**	ddd	-CO-CH=C**H**-
6.16	d	-CO-CH=CH-
6.12	dd	-CO-CH=CH-CH=CH-
5.91	dt	-CO-CH=CH-CH=CH-
2.55	t	-CH$_2$-CO-
2.34	t	-OCO-CH$_2$-
2.30	m	-CH=CH-CH$_2$-
1.32-1.63	m	-CH$_2$-
0.88	t	-CH$_3$

**mKO-c(*E,E*)-dEs** (13-keto-9*E*,11*E*-octadecadienoate; 9-keto-10*E*,12*E*-octadecadienoate)		
Chemical shift (ppm) [a, **]	Multiplicity	Functional group (Type of protons) [*]
**7.13**	dm	-CO-CH=C**H**-
6.14-6.19	m	-CO-CH=CH-CH=CH-
6.07	d	-CO-CH=CH-
2.53	t	-CH$_2$-CO-
2.35	t	-OCO-CH$_2$-
2.17	m	-CH=CH-CH$_2$-
1.32-1.63	m	-CH$_2$-
0.88	t	-CH$_3$

Abbreviations: d: doublet; dd: double doublet; ddd; double of double doublet; t: triplet; dt: double triplet; m: multiplet; dm: double multiplet.

[*] Area of the signals due to the protons in bold, together with the area of the *sn*-1 and *sn*-3 signals of TG shown in Table S1, were used for the quantification of each compound, using the equation [eq. 1] showed in the Materials and Methods.

[**] The assignment of the ^{1}H NMR signals of the protons was made with the aid of standard compounds.

The assignment of the ^{1}H NMR signals of the protons of mKO-c-dEs was made with the data taken from the literature:

[a] Dufour, C.; Loonis, M. (2005). *Chem. Phys. Lipids. 138*(1), 60-68.

Table S10. Chemical shift assignments and multiplicities of ^1H NMR signals in $CDCl_3$ of protons of monoketo monoepoxy *E*-octadecamonoenes (mKO-mEPO-*E*-mEs).

***non vicinal*-mKO-*Z*-mEPO-*E*-mEs**

(13-keto-9,10-*Z*-epoxy-11*E*-octadecenoate; 9-keto-12,13-*Z*-epoxy-10*E*-octadecenoate)

Chemical shift (ppm) [a]	Multiplicity	Functional group (Type of protons) *
**6.66**	dd	-CO-CH=C**H**-
6.40	d	-CO-CH=CH-
3.52	dd	-CH=CH-CHOCH-
3.20	dd	-CH=CH-CHOCH-
2.55	t	-CH$_2$-CO-
2.30	t	-OCO-CH$_2$-
1.2-1.7	m	-CH$_2$-
0.89	t	-CH$_3$

***non vicinal*-mKO-*E*-mEPO-*E*-mEs**

(13-keto-9,10-*E*-epoxy-11*E*-octadecenoate; 9-keto-12,13-*E*-epoxy-10*E*-octadecenoate)

Chemical shift (ppm) [a,**]	Multiplicity	Functional group (Type of protons) *
6.52	dd	-CO-CH=CH-
6.38	d	-CO-CH=CH-
**3.21**	dd	-CH=CH-C**H**OCH-
2.89	td	-CH=CH-CHOCH-
2.53	t	-CH$_2$-CO-
2.30	t	-OCO-CH$_2$-
1.2-1.6	m	-CH$_2$-
0.86	t	-CH$_3$

***vicinal*-mKO-*E*-mEPO-*E*-mEs**

(11-keto-12,13-*E*-epoxy-9*E*-octadecenoate; 11-keto-9,10-*E*-epoxy-12*E*-octadecenoate)

Chemical shift (ppm) [b]	Multiplicity	Functional group (Type of protons) *
**7.02**	dt	-CO-CH=C**H**-
6.16-6.23	dt	-CO-CH=CH-
3.28-3.34	d	-CHOCH-CO-
2.98-3.04	ddd	-CHOCH-CO-
2.29-2.34	t	-OCO-CH$_2$-
2.16-2.22	dtd	-CH=CH-CH$_2$-
1.25-1.70	m	-CH$_2$-
0.82-0.89	t	-CH$_3$

Abbreviations: d: doublet; dd: double doublet; td: triple doublet; ddd: double of double doublet; dtd: double of triple doublet; t: triplet; dt: double triplet; m: multiplet.

* Area of the signals due to the protons in bold, together with the area of the *sn*-1 and *sn*-3 signals of TG shown in Table S1, were used for the quantification of each compound, using the equation [eq. 1] showed in the Materials and Methods.

[**] The assignment of the ^{1}H NMR signals of the protons was made with the aid of standard compounds.
The assignment of the ^{1}H NMR signals of the protons of mKO-mEPO-*E*-mEs was made with the data taken from the literature:
[a] Hidalgo, F.J.; Zamora, R.; Vioque, E. (1992). *Chem. Phys. Lipids. 60*(3), 225-233.
[a,b] Lin, D.; Zhang, J.; Sayre, L.M. (2007). *J. Org. Chem. 72*(25), 9471-9480.
[a] Ramsden, C.E.; Domenichiello, A.F.; Yuan, Z.X.; Sapio, M.R.; Keyes, G.S.; Mishra, S.K.; Gross, J.R.; Majchrzak-Hong, S.; Zamora, D.; Horowitz, M. S.; et al. (2017). *Sci. Sign., 10*(493), eaal5241.
[a] Gardner, H.W.; Kleiman, R.; Weisleder, D. (1974). *Lipids, 9* (9), 696-706.

Table S11. Chemical shift assignments and multiplicities of ^{1}H NMR signals in $CDCl_3$ of protons of different types of aldehydes.

***n*-alkanals**

Chemical shift (ppm) [a,**]	Multiplicity	Functional group (Type of protons) [*]
**9.75**	t	-C$\underline{\mathbf{H}}$O
2.40	dt	CHO-C$\underline{H}_2$-
1.61	m	CHO-CH_2-C$\underline{H}_2$-
1.27-1.32	br	-C$\underline{H}_2$-
0.88	t	-C$\underline{H}_3$

2*E*-alkenals

Chemical shift (ppm) [a,**]	Multiplicity	Functional group (Type of protons) [*]
**9.49**	d	-C$\underline{\mathbf{H}}$O
6.85	tt	CHO-CH=C$\underline{H}$-
6.11	dd	CHO-C$\underline{H}$=CH-
2.32	q	-CH=CH-C$\underline{H}_2$-
1.69-1.19	br	-C$\underline{H}_2$-
0.89	t	-C$\underline{H}_3$

2*E*,4*E*-alkadienals

Chemical shift (ppm) [a,**]	Multiplicity	Functional group (Type of protons) [*]
**9.53**	d	-C$\underline{\mathbf{H}}$O
7.09	m	CHO-CH=C$\underline{H}$-
6.30	m	-CH=C$\underline{H}$-
6.08	dd	CHO-C$\underline{H}$=CH-
2.22	m	-CH=CH-C$\underline{H}_2$-
1.30-1.47	m	-C$\underline{H}_2$-
0.90	t	-C$\underline{H}_3$

4,5-epoxy-2*E*-alkenals

Chemical shift (ppm) [a,**]	Multiplicity	Functional group (Type of protons) [*]
**9.54**	d	-C$\underline{\mathbf{H}}$O
6.56	dd	CHO-CH=C$\underline{H}$-
6.40	dd	CHO-C$\underline{H}$=CH-
3.33	dd	-C$\underline{H}$OCH-
2.96	td	-CHOC$\underline{H}$-
1.33-1.65	m	-C$\underline{H}_2$-
0.91	t	-C$\underline{H}_3$

4-hydroperoxy-2*E*-alkenals

Chemical shift (ppm) [a,**]	Multiplicity	Functional group (Type of protons) [*]

**9.58**	d	-C**H**O
8.20	d	-OOH
6.80	dd	CHO-CH=CH-
6.33	m	CHO-CH=CH-
4.66	dd	-CHOOH-
1.21-1.73	m	$-CH_2-$
0.89	t	$-CH_3$
**4-hydroxy-2*E*-alkenals**		
Chemical shift (ppm) [a,**]	Multiplicity	Functional group (Type of protons) *
**9.57**	d	-C**H**O
6.84	dd	CHO-CH=CH-
6.33	dddd	CHO-CH=CH-
4.43	m	-CHOH-
1.30-1.70	m	$-CH_2-$
0.90	t	$-CH_3$
**2*Z*-alkenals**		
Chemical shift (ppm) [b]	Multiplicity	Functional group (Type of protons) *
**10.06**	d	-C**H**O

Abbreviations: d: doublet; dd:double doublet; td: triple doublet; t: triplet; tt: triple triplet; q: quadruplet; m: multiplet; br: broad signal.

* Area of the signals due to the protons in bold, together with the area of the *sn*-1 and *sn*-3 signals of TG shown in Table S1, were used for the quantification of each compound, using the equation [eq. 1] showed in the Materials and Methods.

** The assignment of the ^{1}H NMR signals of the protons was made with the aid of standard compounds.

The assignment of the ^{1}H NMR signals of the protons of aldehydes was made with the data taken from the literature:

[a] Guillén, M.D.; Ruiz, A. (2004). *Eur. J. Lipid Sci. Tech. 106*(10), 680-687.

[a] Guillén, M.D.; Ruiz, A. (2005a). *Eur. J. Lipid Sci. Tech. 107*(1), 36-47.

[a] Guillén, M.D. ; Ruiz, A. (2005b). *J. Sci. Food Agric. 85*(14), 2413-2420.

[a] Goicoechea, E.; Guillen, M.D. (2010). *J. Agric. Food Chem. 58*(10), 6234-6245.

[b] Moumtaz, S.; Percival, B.C.; Parmar, D.; Grootveld, K.L.; Jansson, P.; Grootveld, M. (2019). *Sci. Rep. 9*, 1-21.

Table S12. Chemical shift assignments and multiplicities of ^{1}H NMR signals in $CDCl_3$ of protons of furan groups.

	**Alkyl-furans**	
Chemical shift (ppm) **	Multiplicity	Functional group (Type of protons) *
7.27	dd	-CH=CH- (ar. C-4)
6.24	dd	-CH=CH- (ar. C-3)
5.94	m	-CH- (ar. C-5)
	**Alkyl furanones (5-pentyl-(5H)-furan-2-one)**	
Chemical shift (ppm) [a]	Multiplicity	Functional group (Type of protons) *
**7.46**	dd	-CH=C**H**- (ar. C-4)
6.10	dd	-CH=CH- (ar- C-3)
5.04	m	-CH- (ar. C-5)
1.26-2.80	m	-CH_2-
0.90	t	-CH_3

Abbreviations: dd: double doublet; t: triplet; m: multiplet.

* Area of the signals due to the protons in bold, together with the area of the *sn*-1 and *sn*-3 signals of TG shown in Table S1, were used for the quantification of each compound, using the equation [eq. 1] showed in the Materials and Methods.

** The assignment of the ^{1}H NMR signals of the protons was made with the aid of standard compounds.

The assignment of the ^{1}H NMR signals of the protons of 5-pentyl-(5H)-furan-2-one was made with the data taken from the literature:

[a] Bonete, P.; Najera, C. (1994). *J. Org. Chem. 59*(11), 3202-3209.

[a] Braukmüller, S.; Brückner, R. (2006). *Eur. J. Org. Chem. 2006*(9), 2110-2118.

Table S13. Chemical shift assignments and multiplicities of ^{1}H NMR signals in $CDCl_3$ of protons of epoxy derivatives coming from linoleic acyl groups.

***Z* epoxides coming from linoleic acyl groups** (Mono-epoxides)		
Chemical shift (ppm) [a,b, **]	Multiplicity	Functional group (Type of protons) *
5.50-5.60	m	-CHOCH-CH$_2$-CH=CH-
5.35-5.50	m	-CHOCH-CH$_2$-CH=CH-
**2.95**	m	-C**H**OC**H**-CH$_2$-CH=CH-
2.20-2.40	m	-CHOCH-CH$_2$-CH=CH-
1.45-1.60	m	-CH$_2$-CH$_2$-CHOCH-
0.87	t	-CH$_3$

***Z* epoxides coming from linoleic acyl groups** (Di-epoxides)		
Chemical shift (ppm) [a, b, **]	Multiplicity	Functional group (Type of protons) *
3.09-3.14	m	-CHOCH-CH$_2$-CHOCH-
3.00	m	-CHOCH-CH$_2$-CHOCH-
1.70-1.85	m	-CHOCH-CH$_2$-CHOCH-
1.45-1.60	m	-CH$_2$-CH$_2$-CHOCH-
0.87	t	-CH$_3$

***E* epoxides coming from linoleic acyl groups** (Mono-epoxides)		
Chemical shift (ppm) [a, **]	Multiplicity	Functional group (Type of protons) *
5.47-5.56	m	-CHOCH-CH$_2$-CH=CH-
5.33-5.42	m	-CHOCH-CH$_2$-CH=CH-
**2.66-2.71**	m	-C**H**O**H**C-
2.35-2.44	m	-CHOCH-CH$_2$-CH=CH-
1.26-1.66	m	-CH$_2$-
0.87	t	-CH$_3$

Abbreviations: t: triplet; m: multiplet.

* Area of the signals due to the protons in bold, together with the area of the *sn*-1 and *sn*-3 signals of TG shown in Table S1, were used for the quantification of each compound, using the equation [eq. 1] showed in the Materials and Methods.

** The assignment of the ^{1}H NMR signals of the protons was made with the aid of standard compounds.

The assignment of the ^{1}H NMR signals of the protons of epoxides derived from linoleic acyl groups was made with the data taken from the literature:

[a] Nilewski, C.; Chapelain, C.L.; Wolfrum, S.; Carreira, E.M. (2015). *Org. Lett. 17*(22), 5602-5605.

[b] Xia, W.; Budge, S.M.; Lumsden, M.D. (2016). *J. Am. Oil. Chem. Soc. 93*, 467-478.

Table S14. Chemical shift assignments and multiplicities of ^{1}H NMR signals in $CDCl_3$ of protons of different types of dihydroxy groups (dHO).

**dHO** (9,10-dihydroxy-12*Z*-octadecanoate)		
Chemical shift (ppm) [a,**]	Multiplicity	Functional group (Type of protons) *
5.52	m	-C<u>H</u>=CH-CH_2-CHOH-CHOH-
5.42	m	-CH=C<u>H</u>-CH_2-CHOH-CHOH-
**3.42**	br	-C<u>**H**</u>OH-C<u>**H**</u>OH-
2.62	br	-CHO<u>H</u>-CHOH-
2.27	t	-OCO-C<u>H</u>$_2$-
2.25	q	-CH=CH-C<u>H</u>$_2$-CHOH-CHOH-
2.02	q	-C<u>H</u>$_2$-CH=CH-CH_2-CHOH-CHOH-
1.28-1.60	br	-C<u>H</u>$_2$-
0.87	t	-C<u>H</u>$_3$

Abbreviations: t: triplet; q:quadruplet; m: multiplet; br: broad signal.

* Area of the signals due to the protons in bold, together with the area of the *sn*-1 and *sn*-3 signals of TG shown in Table S1, were used for the quantification of each compound, using the equation [eq. 1] showed in the Materials and Methods.

** The assignment of the ^{1}H NMR signals of the protons was made with the aid of standard compounds.

The assignment of the ^{1}H NMR signals of the protons of dHO was made with the data taken from the literature:

[a] Nilewski, C.; Chapelain, C.L.; Wolfrum, S.; Carreira, E.M. (2015). *Org. Lett. 17*(22), 5602-5605.

[a] Yang, J.; Morton, M.D.; Hill, D.W.; Grant, D.F. (2006). *Chem. Phys. Lipids. 140* (1-2), 75-87.

Table S15. Chemical shift assignments and multiplicities of ^{1}H NMR signals in $CDCl_3$ of protons of formic acid and formates.

**Formic acid**		
Chemical shift (ppm) [a]	Multiplicity	Functional group (Type of protons) *
**8.01**	s	**H**-COOH
**Formates or polyformates**		
Chemical shift (ppm) [b]	Multiplicity	Functional group (Type of protons) *
**8.05-8.20**	m	-CH_2-CH-OC**H**=O-
2.3	t	-OCO-CH_2-
2.05	s	-CH_2-CH-OCH=O-
1.3-1.5	m	-CH_2-
0.9	t	-CH_3

Abbreviations: s: singlet; t: triplet; m: multiplet.

* Area of the signals due to the protons in bold, together with the area of the *sn*-1 and *sn*-3 signals of TG shown in Table S1, were used for the quantification of each compound, using the equation [eq. 1] showed in the Materials and Methods.

The assignment of the ^{1}H NMR signals of the protons of formic acid and formates was made with the data taken from the literature:

[a] Babij, N. R.; McCusker, E. O.; Whiteker, G. T.; Canturk, B.; Choy, N.; Creemer, L. C.; ... Li, F. (2016). *Org. Process Res. Dev. 20*(3), 661-667.

[b] Harry-O'kuru, R.E.; Biresaw, G.; Tisserat, B.; Evangelista, R. (2016). *J. Lipids, ID 3128604*, 12.
